# The intricate pathogenicity of Group A *Streptococcus*: A comprehensive update

**DOI:** 10.1080/21505594.2024.2412745

**Published:** 2024-10-07

**Authors:** Helena Bergsten, Victor Nizet

**Affiliations:** a Division of Host-Microbe Systems and Therapeutics, Department of Pediatrics, University of California, San Diego School of Medicine, La Jolla, CA, USA; b Department of Microbiology, Tumor and Cell Biology, Karolinska Institutet, Biomedicum, Stockholm, Sweden; c Skaggs School of Pharmacy and Pharmaceutical Sciences, University of California, San Diego, La Jolla, CA, USA

**Keywords:** Group A *Streptococcus*, *Streptococcus* pyogenes, bacterial pathogenesis, virulence, necrotizing fasciitis, toxins

## Abstract

Group A *Streptococcus* (GAS) is a versatile pathogen that targets human lymphoid, decidual, skin, and soft tissues. Recent advancements have shed light on its airborne transmission, lymphatic spread, and interactions with neuronal systems. GAS promotes severe inflammation through mechanisms involving inflammasomes, IL-1β, and T-cell hyperactivation. Additionally, it secretes factors that directly induce skin necrosis via Gasdermin activation and sustains survival and replication in human blood through sophisticated immune evasion strategies. These include lysis of erythrocytes, using red cell membranes for camouflage, resisting antimicrobial peptides, evading phagocytosis, escaping from neutrophil extracellular traps (NETs), inactivating chemokines, and cleaving targeted antibodies. GAS also employs molecular mimicry to traverse connective tissues undetected and exploits the host’s fibrinolytic system, which contributes to its stealth and potential for causing autoimmune conditions after repeated infections. Secreted toxins disrupt host cell membranes, enhancing intracellular survival and directly activating nociceptor neurons to induce pain. Remarkably, GAS possesses mechanisms for precise genome editing to defend against phages, and its fibrinolytic capabilities have found applications in medicine. Immune responses to GAS are paradoxical: robust responses to its virulence factors correlate with more severe disease, whereas recurrent infections often show diminished immune reactions. This review focuses on the multifaceted virulence of GAS and introduces novel concepts in understanding its pathogenicity.

## Introduction

Group A *Streptococcus* (GAS, *S.* *pyogenes)* is frequently carried asymptomatically by school-aged children. It ranks among the top ten causes of infectious disease mortality worldwide [1], exerting a particularly severe impact in developing countries [2]. In wealthier regions, GAS remains a significant health concern due to its capacity to cause a large volume of mild diseases and occasional acute, life-threatening infections in otherwise healthy individuals. Although over a century of research on GAS has elucidated numerous specific virulence factors, it has yet to yield an approved vaccine. While the fundamental clinical features and disease mechanisms of GAS have been extensively reviewed [3,4]; this contribution will focus primarily on recent advancements in understanding the pathogenesis of acute, invasive GAS infections.

## Clinical features

Most children and adults are familiar with strep throat or GAS pharyngotonsillitis, which typically presents as a mild and self-limiting infection that often resolves on its own [5,6]. Similarly, the superficial skin infection impetigo – caused by GAS, *Staphylococcus aureus* (*S*. *aureus*), or both – shares these characteristics. Antibiotic therapy for strep throat is recommended to reduce complications and contagiousness, though it only shortens symptoms by around 16 hours [7].

In many parts of the developing world, recurrent episodes of strep throat significantly increase the risk of acute rheumatic fever (ARF) [8], a condition closely linked to poverty, overcrowded living conditions, and the lack of antibiotic treatment due to insufficient healthcare infrastructure [9]. ARF generally follows strep throat but perhaps also GAS skin infections [10]. Indigenous populations often bear a disproportionate burden of ARF [11]. Recurring ARF episodes heighten the risk of developing rheumatic heart disease (RHD) [8]. RHD remains the principal cause of GAS-related mortality worldwide, affecting 1% of the population in sub-saharan Africa and 1,5% in Oceania, in the final stages requiring surgery to replace or repair heart-valves to prevent heart failure, stroke and death [12]. Glomerulonephritis, an immune complex-mediated condition that can arise from GAS infections of the skin or throat, typically resolves if acute renal complications like fluid overload and hypertension are managed effectively [13].

A less well understood condition linked to GAS is Pediatric Autoimmune Neuropsychiatric Disorders Associated with Streptococcal Infections (PANDAS). Here, neuroinflammation following GAS infections is proposed to trigger symptoms like obsessive-compulsive disorder, tics, and Tourette’s syndrome in children [14]. This disorder shares features with Sydenham’s chorea – affecting 20–30% of ARF patients – which is characterized by involuntary movements and emotional disturbances, and for which a rat model has demonstrated the involvement of antibodies against GAS and the basal ganglia [15]. Recent studies have shown GAS-specific Th17 cells infiltrating the brain in mice after repeated intranasal infections [16], but the association of the symptoms to GAS is still debated. Some argue that Sydenham's chorea and PANDAS are not separate entities but instead should be termed “cerebral rheumatic fever” [17].

GAS can cause severe necrotizing soft tissue infections (NSTI), including necrotizing fasciitis, leading to rapid tissue necrosis and life-threatening conditions. About 30% of NSTI cases occur in previously healthy individuals, with 89% requiring mechanical ventilation within 24 hours due to severe septic shock [18]. GAS is the primary pathogen in monomicrobial NSTI, while other bacteria may cause polymicrobial infections like Fournier’s gangrene. NSTI often results in streptococcal toxic shock syndrome (STSS), characterized by septic shock, multiorgan failure, and toxin effects on skin or mucosal surfaces. Both NSTI and STSS can severely affect children, who often require more intensive care compared to *S. aureus*-induced toxic shock [19]. Despite treatments including surgical debridement, antibiotics, intensive care, IVIG, and hyperbaric oxygen therapy, NSTI remains highly lethal, with an 18% mortality rate and a 22% amputation rate in the largest study to date [18].

In the 19th century, GAS infections were responsible for two out of every three postpartum deaths [20]. Despite advancements in healthcare, GAS puerperal fever remains the leading cause of infection-related death in pregnancy and the puerperium worldwide [21–24]. GAS also significantly affects child health, particularly during scarlet fever epidemics [25], which have seen a resurgence in regions like the UK, Australia [26], China, and Hong Kong [27]. Scarlet fever, a GAS disease characterized by fever, a skin rash (exanthem), and a distinctive bright red “strawberry tongue” [25], was once a leading cause of death among young children. Its impact had diminished in many areas until its recent re-emergence.

GAS infections can manifest in atypical ways that do not align with classical definitions of invasive disease. For example, a notable case involved a young man who developed non-rheumatic GAS myocarditis following a knee abscess. His condition became so severe that it required extracorporeal membrane oxygenation (ECMO) to sustain life [28]. Another unusual case featured a previously healthy pregnant woman who was diagnosed with sinusitis and a subdural empyema, complicated by preeclampsia. After undergoing a cesarean section, she experienced seizures and fell into a coma upon emerging from anesthesia [29].

Over the past decade, there has been a significant increase in scientific research and public health advocacy focused on developing an effective vaccine against GAS [30–33]. Recent advances in GAS research, including a human challenge model for strep throat, are providing crucial insights into pathogenesis and supporting the development of effective vaccines [34,35].

## Prevalence of GAS diseases

**Figure 1.**
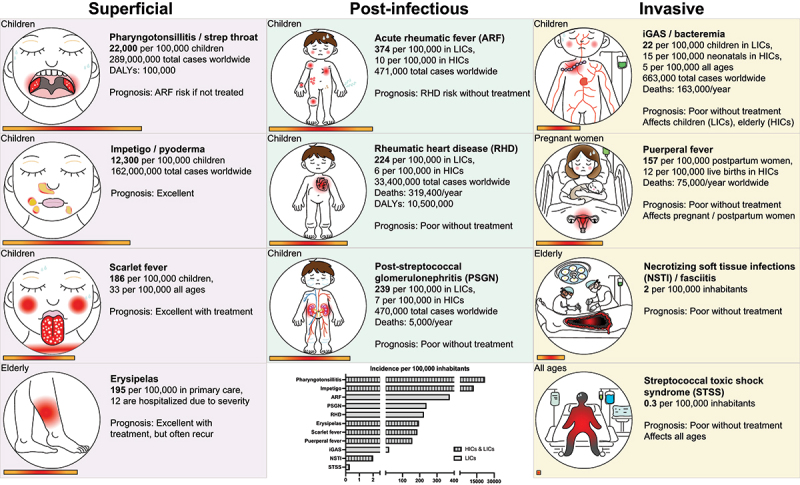
*Epidemiology of Group A Streptococcus (GAS)*. GAS infection induces many common diseases and infection outcomes. The figure indicates the estimated incidence per 100,000 inhabitants and for some diseases prevalence worldwide, deaths/year worldwide and disability-adjusted life years (DALYs)/year worldwide. Estimated incidence in the most affected risk group or setting is displayed. No incidence data was found for cellulitis, a more invasive form of erysipelas, hence not displayed. For NSTI, only GAS cases (30%) are indicated. Graph: note that the incidence scale has been cut to allow for comparisons between less common conditions. The bar below each illustration is proportional to the bars in the graph to allow for incidence comparisons. iGAS: invasive GAS infection, HICs: high-income countries, LICs: low-income countries. References: pharyngotonsillitis [36], impetigo [37], erysipelas [38,39], scarlet fever [25], ARF [1], RHD [12], PSGN [40], iGAS [1,23,24,41], puerperal fever [23,24,42], NSTI [18,43,44], STSS [45,46].

GAS is estimated to cause > 517,000 deaths and > 720 million cases of superficial infections annually worldwide [1]. Estimates of incidence and prevalence of GAS diseases and infection outcomes are found in (Figure 1).

## Acquisition of infection

GAS, a Gram-positive, β-hemolytic, chain-forming bacterium, is primarily a human pathogen with a unique restriction to our species [47]. It is asymptomatically carried in the pharynx by approximately 3% of adults and 8% of school-age children, with these rates exhibiting seasonal peaks during winter. This is when outbreaks frequently occur in schools, with up to 50% of children potentially carrying the outbreak strain without showing symptoms. Notably, children in high-income countries may exhibit the highest rates of GAS carriage [48]. One in every three children experience sore throat every year, and GAS pharyngotonsillitis, or strep throat, account for one in four of those experiences [36]. GAS accounts for 4–10% of pharyngitis cases in adults [1].

**Figure 2.**
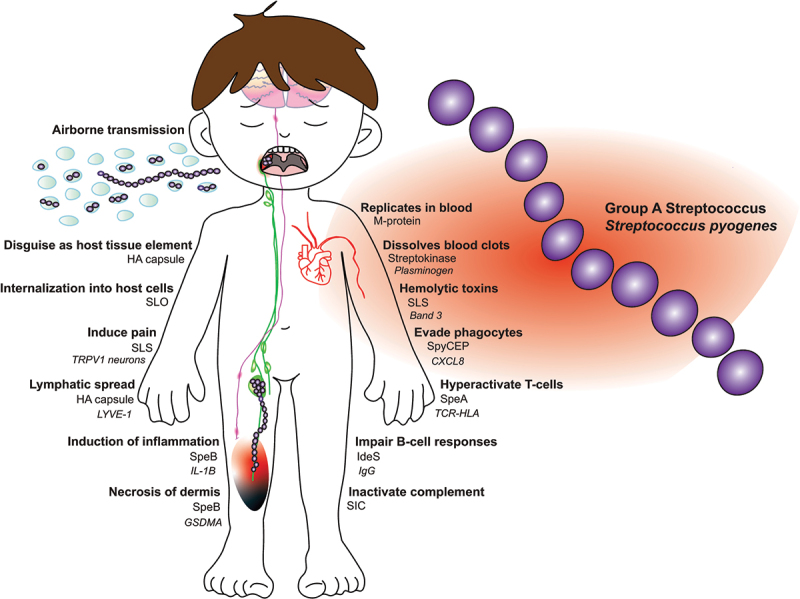
*Clinical features of Group A Streptococcus (GAS)*. GAS exhibits a wide array of pathogenic effects in blood, skin- and soft tissues, the lymphatic system as well as on immune cells and neurons. Clinical aspects are visualized next to an example of a virulence factor with that property and, in some cases, the specific host cell receptor. GAS spread through airborne transmission from asymptomatic pharyngeal carriagers, often school-aged children. In blood, GAS causes hemolysis, replicates, dissolves clots, evades phagocytes, hyperactivates T-cells and impairs B-cell responses. GAS disguises as host tissue elements, internalizes through pore-forming toxins, induces inflammation, pain, dermal necrosis and spreads through lymphatic vessels.

Airborne transmission of GAS, particularly in schools, has been a significant concern since the 1930s-1940s [49,50]. During school-class outbreaks of scarlet fever in the UK, despite hygiene measures, isolation of index cases, and antibiotic treatment, the asymptomatic carriage of the outbreak strain in classroom contacts tripled from the first to the second week [51]. By the third week, 17 to 50% of bacterial settle plates, placed at an elevated location in the classrooms, tested positive for the outbreak strain. Frequent surface disinfection proved ineffective in controlling the spread, as the children themselves were the primary sources of the bacteria. Evidence of heavy asymptomatic shedding (Figure 2), underscores the importance of physical distancing, enhanced respiratory hygiene, and improved ventilation in classrooms during outbreaks [51]. Although scarlet fever mortality can be managed with narrow-spectrum penicillins, the resurgence of the disease and challenges in controlling outbreaks, alongside a concurrent rise in invasive GAS infections, are concerning [52].

Invasive GAS infections can be devastating [41,43]. In the US, between 2005 and 2012, the mortality rates for invasive GAS diseases stood at 11.7%. These rates escalated dramatically for more severe conditions: 29% for NSTI, 38% for streptococcal toxic shock syndrome (STSS), and 45% for patients experiencing septic shock [53]. The reason why GAS leads to mild infections in some but life-threatening conditions in others has been the subject of extensive research. Studies suggest that a higher incidence of invasive disease among household contacts of affected individuals could be due to both the increased virulence of specific GAS strains and shared genetic susceptibility factors [54,55].

Particularly virulent clones, such as the M1T1 clone that arose and disseminated globally in the 1980s, and its sublineage the M1UK that emerged in 2015, are similar to other common GAS strains but have significant molecular advantages [56,57]. The M1T1 clone is an *emm1* (M1) type of GAS and had acquired bacteriophage-encoded DNAse, phage-encoded SpeA, increased expression of SLO and a cytotoxic NAD+ glycohydrolase [58]. The M1UK clone is responsible for the current resurgence of scarlet fever, due to mutations that boosted SpeA expression 10-fold. M1UK swiftly disseminated through the UK [59], as well as in Canada [60] and the US [61], though not New Zealand [62]. In contrast, a scarlet fever outbreak in Hong Kong and China was initially attributed to an *emm12* clone, characterized by antibiotic resistance and a toxic prophage encoding SSA, SpeC, and Spd1 [27]. Subsequent research, however, indicated a significant rise in *emm1* clones, suggesting a more complex epidemiological pattern [63].

Research into the genetic predispositions to invasive streptococcal disease has highlighted the role of HLA alleles. Specific alleles such as *HLA-DRB11501* and *HLA-DQB10602* are associated with a protective effect against severe invasive GAS disease [64], RHD [65,66], and recurrent tonsillitis [67]. Conversely, alleles like HLA-DRB10101 and HLA-DRB10701 are linked to an increased risk of RHD [66,68] and recurrent tonsillitis [67]. A recent case-control study further identified HLA-DQA1 × 01:03 as doubling the risk of invasive GAS disease in otherwise healthy individuals [69]. The interactions between different HLA alleles and the GAS superantigen SpeA also significantly influence disease susceptibility [70]. For instance, HLA-DQA1 interactions with SpeA have been identified as a risk factor for infection [71], while HLA-DQ interactions with SpeA are linked to an increased risk of nasal colonization by GAS [72].

Invasive GAS disease is influenced by a wide array of risk factors. Advanced age may increase susceptibility due to diminished neutrophil responses [73]. Other factors include blunt trauma [74], obesity, diabetes [75], HIV infection, cardiovascular diseases, cancer, injection drug use, residency in long-term care facilities, homelessness, pregnancy, childbirth [22], and having recent influenza [76] or varicella zoster infections [77]. Exposure to children with sore throats also raises the risk [78], as do deficiencies in immune response molecules such as IL-1β [79], IL-6 [80], and IL-17D [81].

NSTI are more commonly associated with blunt trauma, absence of pre-existing skin lesions, and lower BMI compared to non-necrotizing cellulitis [82]. The post-COVID-19 context has also introduced new hypotheses: some suggest that “immune exhaustion” following SARS-CoV-2 infection may predispose individuals to invasive GAS disease, while others attribute the surge in cases to “immune debt,” a result of prolonged social distancing measures [58]. Interestingly, many individuals who develop invasive GAS disease are previously healthy with no known risk factors. Immunity from prior GAS infections can provide some protection, but those who have experienced invasive GAS infections often show lower antibody levels against the M1 protein and superantigens, increasing the risk of future episodes [83].

## Establishment of infection

The establishment of a GAS infection is a complex process that begins with the bacterium attaching to pharyngeal and dermal epithelial cells. In the case of skin infections, areas with previous damage or injury can provide a conduit for GAS to penetrate the dermal barrier. GAS employs a variety of mechanisms to facilitate this attachment, making use of several key molecules (Appendix, Table A1). These include M protein and lipoteichoic acid, which are crucial for initial adherence. Additionally, the bacterium utilizes fibronectin and fibrinogen-binding proteins, collagen-binding proteins, and a hyaluronic acid capsule, all of which enhance its ability to bind firmly to human cells.

GAS exhibits significant genetic diversity, largely due to variations in the M protein, encoded by the *emm* gene. With over 200 distinct *emm* types identified [84], only a select few are responsible for the majority of human infections in high-income countries. GAS strains have historically been categorized as either throat-associated or skin-associated, based on the *emm* types they express. While there is some overlap in the types that cause strep throat and those that lead to invasive disease, there is notably less overlap between the types causing skin infections and those associated with invasive disease [85]. Invasive GAS diseases are most frequently associated with *emm* types 1, 3, 28, and 12, which are linked to severe outcomes like sepsis, STSS, NSTI, and an increased risk of death [53]. These patterns are consistent across different regions, including Europe, where similar *emm* types predominate [86,87]. In China, the most common *emm* types are 12, 1, 3, and 4 [63,88].

**Figure 3.**
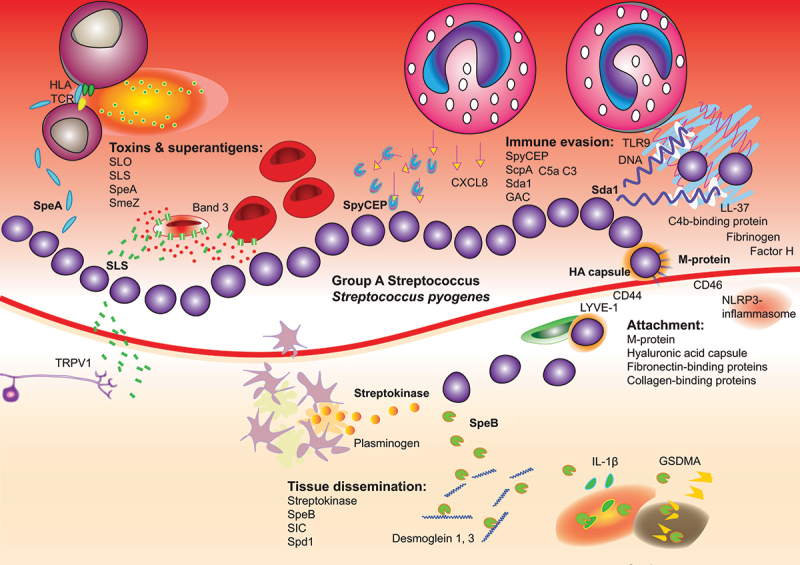
*Pathogenesis of Group A Streptococcus (GAS)*. GAS induces pathology through the actions of toxins and superantigens, immune evasion, host cell attachment, and tissue dissemination. Specific examples of virulence factors in these categories are visualized and, in some cases, the specific host cell receptors. GAS lyses red blood cells, hyperactivate T-cells, evade phagocytes, degrade neutrophil extracellular traps, internalize into host cells and lymphatic vessels, activate the NLRP3 inflammasome and IL-1β, disseminate through host tissues by degrading host proteins through protease activity and fibrinolysis through plasminogen activation. Pain is induced through activation of nociceptor neurons and skin necrosis through gasdermin activation.

The M protein, a pivotal virulence and immunological factor in GAS, was first identified in studies conducted by Rebecca Lancefield a century ago [89]. As the most abundant protein on the GAS surface, it plays a crucial role in the establishment of infection by binding to keratinocytes via the CD46 receptor (Figure 3) [90]. Beyond this initial interaction, M protein significantly contributes to GAS pathogenicity through several mechanisms [86,87], including antigenic variation, inhibition of phagocytosis, blocking the activation of the alternative pathway of the complement system, and exerting a procoagulant effect by inducing the synthesis of tissue factor in endothelial and monocyte cells.

Most GAS strains produce a surface polysaccharide capsule made of hyaluronic acid (HA), which resembles human connective tissue. This capsule is anti-phagocytic and aids in GAS attachment to keratinocytes and pharyngeal cells via the CD44 receptor (Figure 3), causing membrane ruffling and tight junction disruption that facilitate tissue penetration through a paracellular route [91–93]. Synthesis of the HA capsule is orchestrated by the *hasABC* synthase operon [91], which constructs the linear HA polymer by sequentially adding glucuronic acid and β1,3-linked N-acetylglucosamine residues [92,93]. Interestingly, certain invasive GAS serotypes, such as M4 and M12, lack the *hasABC* synthase operon and do not express an HA capsule. Instead, these strains produce HylA, an enzyme that degrades HA in mammalian connective tissues [94]. There is a mutually exclusive expression pattern between the HA capsule and hyaluronidase in GAS strains [94]. Moreover, loss of capsule expression, such as in the pandemic M1T1 clone, is associated with increased expression of an operon encoding toxins [56]. Additionally, the HA capsule contributes to pilus formation and biofilm development [95].

GAS can also co-opt the inflammatory response to its advantage in establishing pharyngeal infection. Specifically, the inflammation induced by interleukin-1 beta (IL-1β) plays a pivotal role by recruiting neutrophils to the nasopharynx. Research indicates that when IL-1β signaling is inhibited, the protease SpeB is neutralized, or neutrophils are depleted, the nasal carriage of GAS is significantly reduced [96]. This suggests that GAS benefits from the inflammatory response in the upper respiratory tract, possibly by disrupting the colonization resistance normally provided by the native microflora [96].

Plasticity is seen in several other GAS proteins than M protein. Historically, the T-antigen has been used as a supplementary serotyping tool to classify GAS strains into 21 T-types. T-antigens are highly variable molecules that make up the GAS pilus and are involved in adhesion, colonization and immune evasion [97]. The mechanisms behind GAS genetic diversity are homologous recombination and high levels of accessory gene plasticity [84]. The maintenance of many distinct genetic lineages of GAS not restricted to geographical boundaries is suggestive of rapid international spread followed by diversifying selection probably driven through immune selection and/or strain competition between phylogroups [84]. Approximately 50% of the accessory gene pool of GAS is phage related [84]. Recent studies of GAS pangenome has systematically analyzed GAS genes *in vitro* and *in vivo* and found that 24% of the genome is essential for GAS survival [98].

Recent studies in non-human primate (NHP) models have identified numerous GAS genes involved in pharyngitis [99], including M protein, ScpA, SOF, and *S. pyogenes* adhesion and division protein (SpyAD), the latter of which also contributed to colonization and disease in NHP studies of GAS genital tract infection [100].

## Immune evasion

A distinctive characteristic of GAS, compared to other major human bacterial pathogens, is its ability to replicate in human blood. This capability is typically assessed using the Lancefield whole blood killing assay [101]. Researchers recognize that the bacterium’s ability to resist opsonization and phagocytosis is a primary factor contributing to its virulence [102]; hence, this capacity remains a subject of extensive research over the years [103].

M protein plays a crucial role in the immune evasion strategies of GAS, primarily by inhibiting the complement system through two key mechanisms. It binds to factor H and fibrinogen (Figure 3), which significantly reduces the opsonization of GAS (Figure 2), thereby impairing the immune system’s ability to mark the bacteria for destruction [104,105]. M protein also interacts with C4b-binding protein to inhibit the classical pathway of complement activation [106]. M protein has affinity for several other plasma proteins, including IgG, IgA and complement regulatory proteins [107]. Different M proteins have different preferences/specificities, e. g. M-type 1, 5 and 6 bind fibrinogen but not C4b-binding protein, which instead is bound by M4, M22 and M60 [108]. Without M protein, GAS becomes highly susceptible to rapid phagocytosis [109]. Immunity developed against a specific strain’s M protein can protect against GAS infection by enhancing phagocytosis and bacterial killing. This has led to the classification of GAS strains into specific M serotypes [110]. However, the immune response to M protein can also have detrimental effects. Antibodies generated against M protein may induce autoimmunity, as they can cross-react with human connective tissue antigens due to structural similarities [111,112]. Additionally, the M gene superfamily extends beyond M proteins to include M-related proteins and immunoglobulin-binding proteins, which also play roles in the bacterium’s interaction with the host immune system [113–115].

The surface-expressed M1 protein of GAS plays a critical role in evading host defenses by interacting with key antimicrobial components. It can sequester and neutralize LL-37, a potent antimicrobial peptide, thereby preventing it from exerting its antibacterial activity [116]. Additionally, M1 protein exhibits resistance to the antimicrobial activity of histones, which are crucial components of neutrophil extracellular traps (NETs). This resistance helps GAS to survive and propagate even in the hostile environment created by neutrophil activation [117].

Invasive M1T1 GAS strains utilize several sophisticated mechanisms to evade neutrophil defenses. These strains express a phage-encoded DNAse, Sda1, which degrades neutrophil extracellular traps (NETs) and the bacterium’s own CpG-rich DNA [118]. Sda1 is critical for promoting resistance to neutrophils and enhancing virulence, as demonstrated in murine models of necrotizing fasciitis [119]. It also suppresses macrophage activity and TLR9-mediated immune responses [119]. Additionally, the streptococcal collagen-like protein 1 (Scl-1), prominently expressed in the M1T1 clone, protects GAS from NET-associated antimicrobial peptides and inhibits the release of myeloperoxidase (MPO), thereby reducing NET formation [120]. GAS can further impair neutrophil defenses by engaging the inhibitory Siglec-9 receptor via its hyaluronic acid (HA) capsule, which blunts the oxidative burst, NET formation, and overall bactericidal activity of neutrophils [121]. The toxin streptolysin O (SLO) also plays a role by impairing neutrophil oxidative burst, degranulation, and NET formation at sublethal concentrations [122].

The classical Lancefield group A carbohydrate (GAC) antigen [123], a high molecular weight polymer consisting of rhamnose with an N-acetylglucosamine (GlcNAc) side chain, makes up about 40–50% of the GAS cell wall. This antigen is the species-defining marker used routinely for the rapid diagnosis of strep throat. Removal of the GlcNAc side chain increases GAS susceptibility to neutrophil killing (Figure 3), platelet-derived antimicrobials in serum, and the antimicrobial peptide LL-37, and results in attenuation in infection models [124]. The impact of the GAC GlcNAc side chain on GAS virulence may vary depending on the presence of other virulence factors within each strain [125]. It is important to note that 2–3% of streptococci that carry the group A antigen are not *S. pyogenes* but rather *Streptococcus dysgalactiae* subspecies *equisimilis* (typically carrying group G or C antigens) or *Streptococcus anginosus* (a commensal viridans streptococcus) [126]. Furthermore, not all M-types of GAS are virulent, and many group G streptococci, which can share pathogenic similarities with GAS, are capable of causing disease [127].

GAS chemokine-inactivating protein (SpyCEP), a surface-bound serine protease, plays a crucial role in immune evasion by targeting and degrading chemokines such as CXCL8 (Figure 3). This activity significantly hinders the recruitment of neutrophils to infection sites (Figure 2), thereby facilitating the persistence and spread of GAS [128,129]. Due to its pivotal role in immune modulation and high immunogenicity, SpyCEP is recognized as an attractive candidate for vaccine development [130]. RNAseq analysis of GAS infected tonsil epithelial cells showed that while GAS infection generally induces a pro-inflammatory response, SpyCEP specifically reduces the levels of CXCL8 post-transcriptionally, thus mitigating one of the body’s key inflammatory reactions to infection [131].

Streptococcal C5a peptidase (ScpA), a surface-bound endopeptidase, plays a critical role in GAS immune evasion by cleaving the complement-derived chemotaxin C5a at its PMN-binding site, thereby inhibiting the recruitment of phagocytic cells (Figure 3). Recent research has further uncovered that ScpA also targets C3, impairing neutrophil activation, phagocytosis, and chemotaxis [132]. Beyond its role in immune modulation, ScpA aids in GAS nasal colonization by adhering to epithelial and endothelial cells through mechanisms independent of the complement system. Moreover, intranasal immunization against ScpA has been shown to prevent GAS infection in murine nasal-associated lymphoid tissue (NALT), highlighting its potential as a target for vaccine development [133].

A recently identified immune evasion strategy employed by GAS involves the use of the surface-associated S protein to capture red blood cell fragments. This mechanism aids GAS survival by cloaking its opsonic targets under natural host cell components, effectively disguising the bacteria from the host immune system. The presence of S protein is crucial for maintaining the virulence of GAS; its absence leads to reduced virulence and affects the development of immunological memory against the pathogen [134].

Although traditionally considered an extracellular pathogen, recent studies have explored the intracellular capabilities of GAS. GAS has demonstrated the ability to survive within macrophages, a process that relies on the presence of M1 protein [135], and is proposed to potentially account for the presence of viable bacteria in biopsies from patients undergoing antibiotic therapy [136]. GAS can replicate within viable human macrophages, indicating an active intracellular life cycle [137]. In the context of pharyngeal keratinocytes, GAS secretes SLO, which triggers autophagy – a process where the cell attempts to digest internalized material. However, the combined actions of SLO and NADase disrupt the maturation of autophagosomes, thereby prolonging GAS’s intracellular survival (Figure 2) [138]. Additionally, GAS evades clearance by keratinocytes due to a lack of ubiquitination, which is essential for targeting bacteria for autophagy [139].

Beyond its classical interactions with phagocytes and lymphocytes, GAS also significantly impacts coagulation and thrombocytes. Severe infections often induce a pro-coagulant and pro-inflammatory state, with some effects directly linked to the actions of M protein [140]. Common complications in severe infections include deep venous thrombosis [141] and thrombocytopenia. These issues may arise from complement activation on the surface of activated thrombocytes, leading to their activation, aggregation into thrombi, and complex formation with neutrophils and monocytes. This process ultimately contributes to thrombocyte phagocytosis [142]. Similarly, the binding of fibrinogen and IgG by GAS in plasma triggers comparable effects, promoting coagulation disturbances [143].

Immunity to GAS is multifaceted and varies significantly with age. Adults generally experience fewer infections than children, partly due to higher antibody titers [144]. Additionally, complete immunity against scarlet fever typically develops over time, with anti-GAS antibodies potentially persisting for up to 45 years. This long duration supports the belief that immunity is primarily M-type specific [145,146]. However, recent studies indicate that invasive GAS infections can elicit both strain-specific and cross-strain specific opsonic antibodies [147]. GAS has evolved sophisticated mechanisms to evade humoral immune responses. For example, IdeS, a cysteine proteinase, specifically cleaves the heavy chain of Immunoglobulin G (IgG), impacting its functionality (Figure 2) [148,149]. Similarly, the endoglycosidase EndoS targets the Fc region of IgG, hydrolyzing it and impairing its effectiveness [150–152]. While IgG antibodies are crucial for protecting against GAS invasion, IgA antibodies play a key role in preventing adherence and colonization [153]. Similar to *S. aureus* protein A, M protein can reverse antibody orientation through Fc-binding, especially in saliva [154]. Additionally, GAS expresses IgA binding proteins that interfere with the effector functions of IgA, further complicating the immune response [155].

While GAS has known evasion strategies for phagocytes, its interactions with other leukocytes like lymphocytes are less documented. Lymphocytes typically mount a Th1-type pro-inflammatory response to GAS, characterized by the production of cytokines such as IL-1β, IL-6, TNF, IL-12, IFN-γ, and IL-18 [156]. Studies on human pharyngitis have shown that individuals who develop pharyngitis exhibit increased serum cytokines, a reduction in conventional lymphocytes, and activation of unconventional lymphocytes [35]. Specifically, after tonsillar challenge, elevated levels of IL-1β, IL-1Ra, IL-6, and IL-18 in saliva, along with IL-1Ra, IL-6, IFN-γ, IP-10, and IL-18 in serum, indicate strong local and systemic pro-inflammatory responses. Additionally, there are increases in classical monocytes and total dendritic cells in peripheral blood, with reductions in B-cells and CD4+ T-cells. While conventional peripheral T-cells show no activation, T-cells expressing γδTCR and Vδ2, as well as MAIT cells, are activated.

## Tissue invasion

In murine models, the histopathology of streptococcal NSTI shows that while the epidermis remains intact initially, the underlying tissues exhibit significant inflammatory infiltrates, pronounced necrosis, and thrombosis, along with a massive bacterial burden, present both as aggregates and within cells [157]. Over time, necrosis progresses from the deeper tissues to the epidermis, demonstrating the GAS’s capability to disseminate through deep layers of soft tissue and invade extensive areas, even with minor epidermal disruptions. The NHP model has been used to identify genes required for necrotizing myositis, and sequencing identified around 100 involved GAS genes [158].

GAS possesses several virulence factors that interact with plasminogen, converting it to plasmin [159,160]. This activation of the host’s clot-dissolving system facilitates the bacterium’s invasion and movement through tissue barriers. Plasmin aids GAS dissemination by proteolytically degrading host defense proteins. Key plasminogen-activating molecules include streptokinase, a well-known secreted thrombolytic enzyme (Figure 2) [161], as well as plasminogen-associated M protein (PAM), alpha-enolase [162], and glyceraldehyde-3-phosphate dehydrogenase. In murine models, blocking streptokinase significantly improves survival rates, likely due to the inhibited ability of GAS to escape from blood clots [163]. Streptokinase activates plasminogen, promoting GAS dissemination at wound sites, as it facilitates the rapid dissolution of fibrin clots and the retraction of the keratinocyte wound layer, thus promoting bacterial spread [164].

Streptokinase is so efficient that it is used in medicine as treatment during thrombo-embolic events such as heart attacks, pulmonary embolisms and arterial clots. There is a production shortage of streptokinase, prompting efforts to genetically engineer less virulent streptococcal strains to increase availability [165]. Despite its significance, GAS can still acquire plasmin without streptokinase by utilizing host activators like urokinase plasminogen activator (uPA) [166], a capability recently demonstrated in a susceptible mouse model [167].

Initially mis-classified as a superantigen, streptococcal pyrogenic exotoxin B (SpeB) is an important virulence factor in GAS (Figure 2). SpeB is both a secreted, extracellular cysteine protease and a surface-bound adhesin with binding activity to laminin and other glycoproteins. The majority of pathogenic GAS strains secrete SpeB [168], which degrades nearly all proteins secreted by GAS, including other virulence factors. This protease plays a vital role in degrading the extracellular matrix, aiding colonization, and disrupting competitor bacteria such as *S. aureus* in biofilms [169]. It also cleaves desmoglein 1 and 3, exacerbating skin involvement in GAS infections [170], and neutralizes the signaling and antibacterial properties of chemokines from inflamed epithelium [171]. SpeB and SIC were among the streptococcal proteins identified in a comprehensive proteomic analysis of GAS infected human samples [172]. Furthermore, SpeB can directly activate IL-1β, bypassing canonical inflammasome pathways, enhancing immune responses that restrict GAS invasion (Figure 3) [79]. In some cases, invasive GAS strains may mutate to repress SpeB expression, helping them evade these immune responses.

Recent studies have linked GAS-induced endothelial apoptosis to both SpeB and the caspase pathway, although the exact mechanisms were initially unclear [173]. A significant breakthrough was the discovery that SpeB triggers epidermal pyroptosis, an inflammatory form of cell death, by cleaving gasdermin A (GSDMA). GSDMA acts as a sensor, substrate, and effector of pyroptosis, making it central to this process (Figure 3) [174,175]. GSDMA cleavage and activation is beneficial in severe GAS infections, as it leads to apoptosis in keratinocytes, protecting mice from widespread disease. Inhibitors of SpeB can enhance GAS clearance in the presence of human neutrophils [176].

The GAS extracellular nuclease Sda1 mediates M1 GAS escape from NETs (Figure 3), and its upregulation *in vivo* serves as a selective force for *covR/S* mutations associated with increased tissue dissemination [177]. Another DNAse, Spd1 (Figure 3), may contribute to nasopharyngeal shedding of GAS. Recent epidemic *emm3* genotype GAS strains are seen to have gained a prophage expressing Spd1 and superantigen SpeC [178].

GAS has a preference for inducing pathology in the lymphatic system and draining lymph nodes. Recent data have enhanced our understanding of GAS lymphatic spread (Figure 2). The lymphatic vessel endothelial receptor-1 (LYVE-1), sharing 41% amino acid sequence similarity with CD44, is identified as a critical host receptor for capsular HA (Figure 3) [179]. Non-encapsulated strains show reduced ability to disseminate to draining lymph nodes *in vivo*, while hyper-encapsulated (mucoid, often *covR/S* mutant) strains have a particular propensity for lymphatics. Recent findings demonstrate GAS spread through lymphatic metastasis: pathogenic spread through lymphatic vessels [180]. The bacteria can enter afferent lymphatics and reach lymph nodes, and use efferent lymphatics to enter the bloodstream [180]. Metastasizing bacteria are extracellular. Furthermore, mild blunt contusion of soft tissue enhances bacterial migration to the local draining lymph node from the site of contusion following GAS bacteremia [181].

Streptococcal inhibitor of complement (SIC) is a virulence factor expressed by M1 strains, known to inactivate components of the complement system (Figure 2, 3), inhibit host antimicrobial factors, and contribute to bacterial adherence to epithelial cells. Human antibodies to SIC are prevalent (around 40%), surpassing the frequency of M1 antibodies [182]. SIC immunization was found to protect mice from disseminating disease following intranasal or intramuscular infection. Notably, naturally occurring SIC antibodies did not provide protection against GAS growth in whole blood, whereas vaccine-induced antibodies did [183].

## Toxins and superantigens

A severe form of streptococcal (or staphylococcal) septic shock is STSS, a state of multiorgan failure and signs of toxicity on mucosal surfaces following infection [184]. STSS also occurs in young, immunocompetent individuals who rapidly deteriorate into life-threatening states with high mortality despite appropriate treatment. The extreme toxicity caused by GAS is likely due to a combination of toxins and superantigens [185]. Another significant manifestation of toxicity associated with GAS is scarlet fever, historically responsible for a significant portion of childhood mortality, now resurgent in many parts of the world.

The pore-forming streptolysin O (SLO) induces rapid, dose-dependent apoptosis in most human cells, particularly macrophages and neutrophils [186]. These cytolysins not only form pores in cholesterol-rich membranes but also exhibit high-affinity lectin activity [187]. SLO promotes survival, replication, and cytosolic growth in macrophages [137]. The recent emergence of pandemic clones of GAS with low capsule expression and high SLO expression has been noted [56]. In human decidual tissues, SLO and SpeB were identified as the main virulence factors [42]. Following intramuscular injections of SLO into rats, decreased tissue perfusion and occlusive intravascular complexes of platelets and neutrophils were observed, indicating that SLO may induce microvascular thrombosis leading to toxin-induced ischemia [188].

Another GAS pore-forming toxin, streptolysin S (SLS), induces a dramatic osmotic change in red blood cells, leading to cell lysis (Figure 2) [189]. Specific binding to ion channels on erythrocytes (Band 3) and keratinocytes (NBCn1) has recently been identified as important (Figure 3) [189,190]. SLS is required for the establishment of nasopharyngeal infections in HLA-transgenic mice and contributes to localized tissue destruction of nasal epithelium [191]. The “pain out of proportion” often described as a hallmark of necrotizing fasciitis may be explained through direct activation of TRPV1+ nociceptor neurons by SLS (Figure 2, 3) [192]. SLS-induced pain triggers the release of a neuropeptide that inhibits recruitment of neutrophils, a form of neuroimmune hijacking. This effect can be blocked both by antagonizing the neuropeptide and through local botulinum toxin (Botox) injection, reducing lesion development and infection-related morbidity.

GAS and *S. aureus* secrete toxins known as superantigens (Figure 3). These molecules cross-link the beta chain (Vβ) of the T-cell receptor with human MHC class II (HLA) expressed on antigen-presenting cells (B cells, monocytes, and dendritic cells). This leads to activation, excessive release of inflammatory cytokines, and proliferation of T-cells (Figure 2). The primary toxin implicated in scarlet fever is thought to be SpeA [25]. In addition to the classical GAS superantigens (SpeA, SpeC, SpeG-M, SmeZ, and SSA), two new superantigens have been recently described: SpeQ and SpeR [193]. These 13 superantigens are expressed in different combinations and quantities by different clinical isolates, as the majority of them are encoded on mobile genetic elements. A typical GAS isolate expresses 3–4 superantigens, with SpeA being the most common to find in NSTI or STSS isolates [194]. Even though superantigens are highly effective at low concentrations, the quantity of expression can matter. The expression of SpeA was 9 times higher in the M1UK clone than in comparable isolates [59]. However, no correlation has been demonstrated between the amount of superantigen expressed and disease severity; rather, the opposite is observed. Identical strains can cause both mild and severe invasive disease [195]. Individuals with a propensity to respond more strongly to superantigens develop more severe manifestations [196], and low humoral immunity confers susceptibility to severe disease [83].

In addition to severe disease and cytokine storms, superantigen function is linked to GAS colonization [72]. Nasopharyngeal infection by GAS requires superantigen-responsive Vβ-specific T cells, suggesting that GAS manipulate T-cells to establish nasopharyngeal infection [197]. Thus, superantigen interactions with host cells not only depend on HLA [64], but also the Vβ-profile of the host’s T-cell repertoire. Typically, superantigen activation results in the expansion of T-cells with specific Vβ receptors in the acute phase, followed by depletion of that specific T-cell population [19,198]. Vβ activation in STSS correlates with the number of organ dysfunctions [19]. A newly described subset of T-cells, the mucosal-associated invariant T-cells (MAIT-cells), has been linked to superantigen activation. Although few in circulation, MAIT cells are the main responders among T-cells to superantigens and produce a majority of the cytokines [199]. MAIT cells in patients with STSS were activated and proliferated. In tonsillar tissue, the presence of SpeA and other superantigens resulted in B cell apoptosis and abrogation of total IgA, IgM, and IgG production [200]. The superantigens drove the follicular T-cells to a proliferating phenotype with the loss of tonsillar B-cells and antibody production.

M protein is anchored to the cell wall of GAS by sortase A [201]. While not generally considered a superantigen, M protein exhibits superantigen activity when in a highly purified soluble form lacking the membrane-spanning region [202]. In circulation, the virulence contributions of M protein expand from phagocyte evasion to additional potent proinflammatory effects. M protein released from the bacterial surface forms pathological complexes with fibrinogen [83], leading to the activation of neutrophils through beta2 integrins [84]. This activation results in the release of heparin-binding protein, which induces vascular leakage and contributes to severe pulmonary damage and multi-organ failure, characteristics of STSS [84]. Released M1 protein also activates the NLRP3 inflammasome, leading to the release of IL-1β and macrophage programmed cell death [203].

## In tissue-persistence: Intracellular survival, biofilm and immune modulation

Streptococci are well-known to respond appropriately to narrow-spectrum antibiotics, such as simple penicillins. However, recurrent disease, indicative of local persistence, is well-known clinically [204], and biopsies from severe tissue infections such as NSTIs contain a high bacterial burden despite prolonged antibiotic treatment [136]. Recurrence of erysipelas is around 16% [205]. Intracellular invasion by GAS, once considered an extracellular bacterium, was described long ago [153,206]. Tonsils excised from individuals after treatment failure of pharyngotonsillitis harbor intracellular GAS [207]. Fibronectin-binding proteins associated with internalization are similar between invasive and non-invasive isolates [208]. This internalization might represent successful containment by the host, but it could also lead to invasion of deeper tissues or constitute a pathogen reservoir with associated risk of recurrence. Another possible explanation for persistence in GAS infections is biofilm formation, identified in tissue biopsies from a third of NSTI patients [209]. Biofilm formation can be directly influenced by host and environmental factors [210].

GAS preferentially targets human tonsils, akin to nasal mucosa-associated lymphoid tissue (NALT) in mice [133]. GAS stimulation results in the expansion of CD4+ IL-17+ T-cells in NALT [211]. Exposure to saliva leads to GAS aggregation and inhibits binding to buccal epithelium [212]. Recurring tonsillitis, linked to immunosusceptibility involving HLA haplotypes and follicular T-cells, shows reduced germinal center size and fewer helper T-cells and B-cells, impairing antibody responses [67]. In recurrent tonsillitis, germinal center T-cells express granzyme B, leading to B-cell cytotoxicity, revealing a novel host-pathogen interaction mechanism.

## Genetics and the regulation of virulence

The GAS genome, spanning around 1.85 Mb and encompassing approximately 1,800 genes, contains many genes whose roles in pathogenesis are still not fully understood [213]. Key to the coordinated expression of these genes are several genetic regulatory systems, which include response regulators and two component signal transduction systems [214]. The response regulators, such as multiple gene regulator (mga), RofA-like protein (RALP), and Rgg/RopB, control expression of various virulence factors in a growth phase-dependent manner. The mga regulator, for instance, activates the transcription of multiple virulence factors such as M protein, ScpA, M-like proteins, serum opacity factor (SOF) and SIC [3]. Conversely, Nra (negative regulator of GAS) suppresses these and other genes, including mga itself [215]. Two-component systems such as CsrRS/CovRS, FasBCAX, and Ikk/Irr play crucial roles in modulating GAS pathogenicity. The CovRS system, in particular, represses the expression of about 15% of the GAS transcriptome, including many virulence factors [216], and its inactivation can lead to increased virulence. This system is a known hotspot for inactivating mutations that can enhance GAS pathogenicity [216]. The Ihk/irr two component system is upregulated during pharyngitis [217], promotes evasion of neutrophil phagocytosis, and is required for full virulence in a mouse infection model [218]. Ihk/Irr is likewise transiently upregulated in GAS shortly following intracellular uptake in macrophages; however, after several hours, up-regulation of the CovR/S system predominates [219].

A new quorum-sensing system, sil, present in a subpopulation of GAS strains, controls the expression of bacteriocins in response to host signals like asparagine [220]. This system can trigger an autoinduction mechanism that gives GAS a competitive advantage in polymicrobial environments. Additionally, GAS induces endoplasmic reticulum stress to acquire asparagine, a process that can be mitigated by PERK/ISR inhibitors, which have shown promise in reducing bacterial load and tissue damage in the infected host [221]. Furthermore, GAS adapts to various environmental stressors, such as glucose starvation, by upregulating the arginine deiminase pathway. This pathway enhances bacterial survival and virulence, partly by increasing the expression of exotoxins [222]. In polymicrobial environments, the Gram-negative metabolite Oxo-C12 has been shown to promote GAS adherence to host tissues and biofilm formation, highlighting the complex interplay between GAS and other microbial communities [223].

GAS is a natural source of Cas9 nuclease [224], often used today as a genome editing-tool for precise DNA targeting [225,226]. Cas9 is considered a bacterial immune defense against phages and plasmids, but it is increasingly recognized as a GAS virulence factor. Cas9 mediates adherence, growth in human blood, and virulence in a murine NSTI model [227]. Most humans have antibodies [228] and T-cell responses to Cas9 [229]. The GAS CRISPR-Cas9 system prioritizes defense against the most recent invader [230]. Additionally, there are certain phages that neutralize this bacterial immune system [231,232].

## Diagnostic issues of invasive disease

Diagnosing NSTI, including necrotizing fasciitis and myositis, presents challenges due to the absence of cardinal symptoms. The most common symptom observed is bruising of the skin in 51% of patients [18]. Other frequent symptoms include severe pain requiring opioids (42%), purple/black discoloration of the skin (32%), gas on radiology (30%), skin bullae (27%), crepitus (14%) and skin anaesthesia (6%). However, the majority of patients (87%) exhibit one or more of these findings. Although laboratory values suggest infection, no specific biomarker for NSTI has been identified.

The Laboratory Risk Indicator for Necrotizing Fasciitis (LRINEC) score was developed to predict NSTI risk early on [233], but its predictive value is limited [234]. Efforts to enhance the LRINEC score by emphasizing high CRP values and clinical features like pain out of proportion have been made [235]. Some advocate for rapid StrepA testing in NSTI cases [236] as in pharyngitis [237]. Other investigated biomarkers include the nitric oxide system [238] and inflammatory cytokines [234]. Pentraxin-3 has been associated with negative outcomes such as septic shock, amputation, and risk of death [239]. Thrombomodulin has been proposed as a biomarker for NSTI, showing promise in discriminating between NSTI and non-NSTI cases [240]. Additionally, a distinct biomarker profile distinguishing GAS NSTI from other types of NSTI has been identified, involving differential expression of IL-2, IL-10, IL-22, CXCL10, Fas-ligand, and MMP9.

Proteomic analysis of NSTI samples identified 19 GAS proteins, including SIC, trigger factor (TF), and phosphoglycerate kinase [172]. Among human proteins detected, 38% were neutrophil proteins, such as alpha enolase and lactotransferrin, proposed as biomarkers. Transcriptomic analysis revealed a strong interferon-related response specific to GAS NSTIs, with mediators CXCL9, CXCL10, and CXCL11 identified as potential diagnostic biomarkers [241]. Further technical advances promise to improve our understanding of the landscape of proteins at work during streptococcal infections. Detailed mass spectrometry of human plasma protein interactions with GAS found both already described virulence mechanisms and new interactions [242]. Detailed proteomics of mice infected by GAS found markers trackable in plasma samples of infected patients [243].

Diagnosing STSS can be challenging, despite consensus definitions [184]. It is possible that physicians frequently categorize STSS cases as septic shock or invasive GAS disease because of the overlap in presentation such as hypotension with multiple organ failure and isolation of GAS from a normally sterile site, and STSS could be 5.3 times more common than what is currently diagnosed by US physicians [45]. Importantly, there is no age restriction in the STSS criteria.

## Treatment of invasive infections

Most GAS infections are treatable with penicillin [237], but treatment of invasive disease requires more complex management. A standard approach combines a β-lactam antibiotic with clindamycin to reduce toxin production, alongside surgical debridement. Despite these measures, mortality persists, leading physicians to consider additional treatments, though their efficacy is uncertain. In a large NSTI patient cohort, all 409 patients underwent surgery within a median of 19 hours of admission, with a median of 4 surgeries [18]. Most patients received combination antibiotics: 98% clindamycin, 87% a carbapenem, and 62% ciprofloxacin. Additionally, 80% received hyperbaric oxygen treatment (HBOT) and 58% received intravenous immunoglobulin (IVIG). The effectiveness of HBOT and IVIG in treating GAS NSTI remains debated.

The efficacy of adjunctive clindamycin treatment can be related to reduced Sda1 and SLO activity [244]. A retrospective cohort study of US hospitals showed that adjunctive clindamycin confers a mortality benefit [245]. The odds ratio for in-hospital mortality was 0.44 for clindamycin treated patients with invasive GAS infections, compared to non-clindamycin treated patients.

IVIG treatment confers inhibitory activity against superantigens [246–248], thus it was proposed that IVIG could enable a conservative surgical approach in combination with clindamycin [249]. A single-center randomized controlled study of adjunctive IVIG in NSTI of all microbiological etiologies showed no benefit [250]. However, in the subgroup of patients dominated by GAS infections, i.e. those patients with NSTI of the extremities, IVIG treatment was found to be beneficial. A single dose of 25 g IVIG is sufficient to achieve superantigen neutralization, and there is a correlation between administered dose IVIG and superantigen protection [194]. IVIG was associated with survival in a cohort of 126 patients with GAS NSTI [82]. In STSS, IVIG is a clear survival factor, shown in this meta-analysis [251]. Recently, affinity purification of IVIG was shown to increase its effectiveness in promoting GAS opsonophagocytosis [252].

In the case of HBOT, a systematic review from 2005 failed to locate any relevant clinical evidence supporting or refuting its effectiveness in managing NSTI [253]. Randomized trials are sorely needed. However, a meta-analysis of non-randomized studies involving patients with NSTI of all microbiological etiologies showed a pooled odds ratio for in-hospital mortality of 0.44 in favor of HBOT [254]. Further, the odds ratio for amputation was 0.6 in favor of HBOT, and patients ineligible for HBOT (e.g. due to severe hemodynamic instability) showed decreased odds of survival [255]. An American nationwide retrospective study involving 60,481 patients with NSTI of all microbiological etiologies revealed that HBOT is associated with decreased mortality and amputations, despite the fact that only < 1% of patients received it between 2012 and 2020 [256].

Finally, interesting preclinical research is beginning to emerge in the realm of specific anti-virulence therapeutic strategies targeting GAS. For instance, treatment with a pan-caspase inhibitor reduced GAS skin lesion size and bacterial counts in mice [257], and similar reduction in skin lesion size occurred when GAS-induced pain was blocked by local Botox injection [192]. Additional examples include the broad-spectrum neutralization of GAS pore-forming toxins achieved with human erythrocyte membrane-coated nanoparticles [258], the development and characterization of a SpeB inhibitor [176], and monoclonal antibodies against SLO or M protein that reduced morbidity in GAS superinfection of influenza in a murine model [259].

## Antibiotic resistance

A retrospective study of American NSTI patients between 2015–2018 found that clindamycin resistance was common (31%) in GAS NSTI isolates, and that this resistance was associated with more frequent need for amputations [260]. Adjunctive clindamycin may be replaced by linezolid as resistance to clindamycin increases [261]. Another alternative could be tedizolid (a newer oxazolidinone) that had comparable results to linezolid in a murine GAS model [262]. Additionally, adjunctive treatment with rifampicin could be added to the β-lactam and clindamycin regimen [263]. A GAS-infected skin tissue model showed surprisingly high bacterial counts after treatment with penicillin and clindamycin in high doses, while adjunctive rifampicin reduced bacterial counts and bacterial metabolism. Concerningly, the first step in developing β-lactam resistance has been discovered in two GAS strains [264]. A study of 7025 GAS genomes identified 137 strains with reduced β-lactam MICs [265]. This is a warning signal about a potential future where strep throats may not be so reliably treated with penicillin, with massive clinical implications.

Antibiotic resistance has also been described in GAS against commonly used antibiotics such as erythromycin, tetracycline and fluoroquinolone [266–268]. On a global scale, 38% of GAS strains are tetracycline resistant and 25% erythromycin resistant [267]. During a scarlet fever outbreak in Beijing, 96% of strains were erythromycin resistant, 94% tetracycline resistant and 79% were clindamycin resistant [266].

## Progress in GAS vaccine development

Lack of relevant animal models, high genetic diversity of antigen targets, safety concerns, lack of consensus on clinical endpoints for establishment of proof of concept, and uncertain market incentives have created major impediments to progress in GAS vaccine development [269]. The pipeline of GAS vaccines remains relatively empty [270]. However, the field of GAS vaccine development has had a revival, especially since the 2018 WHO resolution on rheumatic fever and RHD.

The most advanced M protein (StreptAnova 30-valent, J8/S2 combivax, P * 17/S2 combivax, StreptInCor) and non-M protein (Combo4, VAX-A1, Combo5, TeeVax) vaccine candidates are reviewed in [270]. Successful early phase clinical trials in humans have been conducted without serious safety signals with 4 M protein vaccine candidates: a 6-valent, a 26-valent, a 30-valent (all N-terminal) and a conserved C-repeat region M protein vaccine [271–274]. The 6-valent vaccine provided the first evidence in humans that hybrid fusion M protein is a feasible strategy [271]. The 26-valent [272] and 30-valent [273] vaccine expanded the technique and the C-repeat region vaccine showed that immunity against more conserved regions of the M protein is possible and safe [274].

In the non-M protein based arena, the Combo5 vaccine uses antigens SLO, SpyCEP, ScpA, arginine deiminase (ADI), and TF. It has been tested in the NHP-model, where antibody responses against all antigens were detected in serum and immunized NHPs showed a reduction in pharyngitis and tonsillitis [275]. Another non-M protein vaccine candidate named VAX-A1 uses modified GAC conjugated to SpyAD in combination with SLO and ScpA [276]. This has been developed further using non-native amino acid click-chemistry to conjugate GAC to SLO which successfully generated functional antibodies and protected mice against systemic GAS challenge [277]. The TeeVax vaccine candidate focus on T-antigens [97] and GlaxoSmithKline/GVGH has a non-M protein combination vaccine consisting of SpyCEP, SLO, SpyAD recombinant proteins and native GAC conjugated to a carrier protein [278].

Immunization to the conserved (J8) region of the M protein and to the superantigen SpeC protect mice against STSS [279]. Also, passive immunotherapy with antibodies to J8 could resolve established disease, which could be further enhanced by addition of SpeC antibodies.

An opsonophagocytic killing assay has been developed to measure serum protection against GAS without fresh neutrophils and complement, to reduce donor variability [280]. The assay utilizes human promyelocytic leukemia cells as a source of neutrophils and baby rabbit complement, giving the model a potential to provide a robust and reproducible platform to accelerate vaccine development. However, an opsonophagocytosis assay alone might be inadequate to understand protection after GAS vaccination, particularly considering protection from immune evasion factors [281]. A recent advancement has been the development of the human infection model of pharyngitis [282]. During 2018–2019 a total of 25 healthy adults were challenged with GAS and pharyngitis was diagnosed in 85% at the starting dose level (1-3×10^5 CFU/mL). Antibiotic treatment was started at diagnosis of pharyngitis or at 5 days post-challenge. This model can be used to establish immune correlates for protection to help vaccine development.

## Outlook

GAS produces a broad repertoire of virulence factors, and we are getting closer to answering which of them are key to driving pathogenesis. However, this represents a race against the clock, since new strains arise and the molecular evolutionary events transpiring in just one bacterial cell can ultimately spread and produce millions of human infections worldwide [57]. During the initial year of the COVID-19 pandemic, invasive GAS infections decreased rapidly, showing that protection against disease is possible. Today though, invasive GAS disease is back, and we are seeing record-breaking numbers of infections in many countries, a critical opportunity to conduct patient-based research. When clinical awareness is high, and all available therapy is given, mortality in NSTI is still around 18% and the amputation rate is 22% [18]. There is a substantial burden of invasive GAS disease in pregnancy and young children in low-income countries [24] and 319,000 people die from RHD every year [12]. With new drugs, this could decrease, but the threat of antibiotic resistance is approaching and becoming real among additional streptococcal pathogens, indicating that clinical outcomes could actually deteriorate. For example, centers located in high clindamycin-resistant areas may be advised to adjust to linezolid as adjunctive treatment in NSTI. The future threat of β-lactam resistant GAS exists, but is balanced by the advances in vaccine development. Increasing numbers of potential therapeutics and vaccines are in the pipeline, yet while waiting, we should aim to reduce barriers to access primary healthcare [283].

## Supplementary Material

240808_GAS_virulence_factors_Table -S1.xlsx

## Data Availability

Data sharing is not applicable to this article as no new data were created or analyzed in this study.

## A1 Appendix

**Table A1.** GAS virulence factors.

Virulence factor	Figure	References
M protein	1, 2	[45,76,108,109,127,128,132,134,135,195,274,284]
HA capsule	1, 2	[48,83,86,87,113,171–173,285]
Fibronectin-binding protein	2	[201]
Collagen-binding protein	2	
SLO	1, 2	[48,114,129,130,179–181]
SLS	1, 2	[182,183,185]
SpeA	1, 2	[64,75,187–190,192,193]
SpeB	1, 2	[71,88,160–164,166–168]
SpeC, SpeD, SpeF, SpeG, SpeH, SpeJ, SpeK, SpeL, SpeM, SpeQ, SpeR, SSA		[186]
SmeZ	2	
SpyCEP	1, 2	[120–123]
ScpA	2	[124,125]
SpnA		
Scl-1	2	[112]
Streptokinase	2	[151–153,155]
Sda1	2	[110,111]
Spd1	2	
DNAse		
NADase		[130,286]
LTA		
Fibronectin		
GAC	2	[116,117]
SIC	1, 2	[164,174,175]
S protein		[126]
IdeS	1	[140,141]
EndoS		[142–144]
Alpha-enolase		[154,164]
uPA		[158,159]
SpyAD		[91,92]
Sil		[213]
Oxo-C12		[216]
Arginine deaminase		

## References

[1] Carapetis JR, Steer AC, Mulholland EK, et al. The global burden of group A streptococcal diseases. Lancet Infect Dis. 2005;5(11):685–26. doi: 10.1016/S1473-3099(05)70267-X 16253886

[2] Good MF, Weinberger DM. *Streptococcus*: an organism causing diseases beyond neglect. PloS Negl Trop Dis. 2020;14(5):e0008095. doi: 10.1371/journal.pntd.0008095 32437344 PMC7241690

[3] Cunningham MW. Pathogenesis of group A streptococcal infections. Clin Microbiol Rev. 2000;13:470–511. doi: 10.1128/CMR.13.3.470 10885988 PMC88944

[4] Walker MJ, Barnett TC, McArthur JD, et al. Disease manifestations and pathogenic mechanisms of group A Streptococcus. Clin Microbiol Rev. 2014;27(2):264–301. doi: 10.1128/CMR.00101-13 24696436 PMC3993104

[5] Centor RM, Meier FA. Diagnostic decision: throat cultures and rapid tests for diagnosis of group A streptococcal pharyngitis. Ann Intern Med. 1986;(6):892. doi: 10.7326/0003-4819-105-6-892 3535604

[6] Barnett ML, Linder JA. Antibiotic prescribing to adults with sore throat in the United States, 1997-2010. JAMA Intern Med. 2014;174(1):138–140. doi: 10.1001/jamainternmed.2013.11673 24091806 PMC4526245

[7] Spinks A, Glasziou PP, Del Mar CB. *Antibiotics for sore throat*, cochrane database syst. Rev. 2013, pp. CD000023. 10.1002/14651858.CD000023.pub4 PMC6457983 24190439

[8] Carapetis JR, McDonald M, Wilson NJ. Acute rheumatic fever. The Lancet. 2005;366(9480):155–168. doi: 10.1016/S0140-6736(05)66874-2 16005340

[9] Karthikeyan G, Guilherme L. Acute rheumatic fever. The Lancet. 2018;392(10142):161–174. doi: 10.1016/S0140-6736(18)30999-1 30025809

[10] Thomas S, Bennett J, Jack S, et al. Descriptive analysis of group A Streptococcus in skin swabs and acute rheumatic fever, Auckland, New Zealand, 2010–2016. The Lancet Reg Health - West Pac. 2021;8:100101. doi: 10.1016/j.lanwpc.2021.100101 34327427 PMC8315459

[11] Wyber R, Wade V, Anderson A, et al. Rheumatic heart disease in indigenous young peoples. Lancet Child Adolesc Health. 2021;5(6):437–446. doi: 10.1016/S2352-4642(20)30308-4 33705693

[12] Watkins DA, Johnson CO, Colquhoun SM, et al. Global, regional, and national burden of rheumatic heart disease, 1990–2015. N Engl J Med. 2017;377(8):713–722. doi: 10.1056/NEJMoa1603693 28834488

[13] Alhamoud MA, Salloot IZ, Mohiuddin SS, et al. A comprehensive review study on glomerulonephritis associated with post-streptococcal infection. Cureus. 2021;13:e20212. doi: 10.7759/cureus.20212 35004032 PMC8730744

[14] Leckman JF, King RA, Gilbert DL, et al. 4th Streptococcal upper respiratory tract infections and exacerbations of tic and obsessive-compulsive symptoms: a prospective longitudinal study. J Am Acad Child Adolesc Psychiatry. 2011;50(2):108–118.e3. doi: 10.1016/j.jaac.2010.10.011 21241948 PMC3024577

[15] Brimberg L, Benhar I, Mascaro-Blanco A, et al. Behavioral, pharmacological, and immunological abnormalities after streptococcal exposure: a novel rat model of Sydenham chorea and related neuropsychiatric disorders. Neuropsychopharmacology. 2012;37(9):2076–2087. doi: 10.1038/npp.2012.56 22534626 PMC3398718

[16] Dileepan T, Smith ED, Knowland D, et al. Group A *Streptococcus* intranasal infection promotes CNS infiltration by streptococcal-specific Th17 cells. J Clin Invest. 2016;126(1):303–317. doi: 10.1172/JCI80792 26657857 PMC4701547

[17] Malmborg P, Dahlström K, Kendahl GC, et al. Rheumatic fever behind acute chorea in a young girl. A case report. Lakartidningen. 2001;98(34):3545–3549. 11571798

[18] Madsen MB, Skrede S, Perner A, et al. Patient’s characteristics and outcomes in necrotising soft-tissue infections: results from a Scandinavian, multicentre, prospective cohort study. Intensive Care Med. 2019;45(9):1241–1251. doi: 10.1007/s00134-019-05730-x 31440795

[19] Javouhey E, Bolze P-A, Jamen C, et al. Similarities and differences between staphylococcal and streptococcal toxic shock syndromes in children: results from a 30-case cohort. Front Pediatr. 2018;6:360. doi: 10.3389/fped.2018.00360 30547021 PMC6280580

[20] Gergis H, Barik S, Lim K, et al. Life-threatening puerperal infection with group a streptococcus. J R Soc Med. 1999;92(8):412–413. doi: 10.1177/014107689909200811 10656011 PMC1297321

[21] Sriskandan S. Severe peripartum sepsis. J R Coll Physicians Edinb. 2011;41(4):339–346. doi: 10.4997/JRCPE.2011.411 22184573

[22] Hamilton SM, Stevens DL, Bryant AE. Pregnancy-related group a streptococcal infections: temporal relationships between bacterial acquisition, infection onset, clinical findings, and outcome. Clin Infect Dis. 2013;57(6):870–876. doi: 10.1093/cid/cit282 23645851 PMC3749745

[23] Leonard A, Wright A, Saavedra-Campos M, et al. Severe group a streptococcal infections in mothers and their newborns in London and the South East, 2010–2016: assessment of risk and audit of public health management. BJOG. 2019;126(1):44–53. doi: 10.1111/1471-0528.15415 30070056

[24] Sherwood E, Vergnano S, Kakuchi I, et al. Invasive group a streptococcal disease in pregnant women and young children: a systematic review and meta-analysis. Lancet Infect Dis. 2022;22(7):1076–1088. doi: 10.1016/S1473-3099(21)00672-1 35390294 PMC9217756

[25] Lamagni T, Guy R, Chand M, et al. Resurgence of scarlet fever in England, 2014–16: a population-based surveillance study. Lancet Infect Dis. 2018;18(2):180–187. doi: 10.1016/S1473-3099(17)30693-X 29191628

[26] Walker MJ, Brouwer S, Forde BM, et al. Detection of epidemic scarlet fever group a streptococcus in Australia. Clin Infect Dis. 2019;69(7):1232–1234. doi: 10.1093/cid/ciz099 30721938

[27] You Y, Davies MR, Protani M, et al. Scarlet fever epidemic in China caused by Streptococcus pyogenes serotype M12: epidemiologic and molecular analysis. EBioMedicine. 2018;28:128–135. doi: 10.1016/j.ebiom.2018.01.010 29342444 PMC5835554

[28] Fox-Lewis A, Merz TM, Hennessy I. Severe non-rheumatic streptococcal myocarditis requiring extracorporeal membrane oxygenation support. Lancet Infect Dis. 2020;20(12):1481. doi: 10.1016/S1473-3099(20)30689-7 33248041

[29] Poignant S, Pigache P, Fournier E, et al. Streptococcus pyogenes subdural empyema and pre-eclampsia. Lancet Infect Dis. 2018;18(4):473. doi: 10.1016/S1473-3099(18)30096-3 29582773

[30] Dale JB, Walker MJ. Update on group a streptococcal vaccine development. Curr Opin Infect Dis. 2020;33(3):244–250. doi: 10.1097/QCO.0000000000000644 32304470 PMC7326309

[31] Schödel F, Moreland NJ, Wittes JT, et al. Clinical development strategy for a candidate group a streptococcal vaccine. Vaccine. 2017;35(16):2007–2014. doi: 10.1016/j.vaccine.2017.02.060 28318768

[32] Moore HC, Cannon JW, Kaslow DC, et al. A systematic framework for prioritizing burden of disease data required for vaccine development and implementation: the case for group a streptococcal diseases. Clin Infect Dis. 2022;75(7):1245–1254. doi: 10.1093/cid/ciac291 35438130 PMC9525082

[33] Andrejko K, Whittles LK, Lewnard JA. Health-economic value of vaccination against group a streptococcus in the United States. Clin Infect Dis. 2022;74(6):983–992. doi: 10.1093/cid/ciab597 34192307

[34] Osowicki J, Azzopardi KI, Baker C, et al. Controlled human infection for vaccination against Streptococcus pyogenes (CHIVAS): establishing a group a Streptococcus pharyngitis human infection study. Vaccine. 2019;37(26):3485–3494. doi: 10.1016/j.vaccine.2019.03.059 31101422

[35] Anderson J, Imran S, Frost HR, et al. Immune signature of acute pharyngitis in a Streptococcus pyogenes human challenge trial. Nat Commun. 2022;13(1):769. doi: 10.1038/s41467-022-28335-3 35140232 PMC8828729

[36] Miller KM, Carapetis JR, Van Beneden CA, et al. The global burden of sore throat and group a streptococcus pharyngitis: a systematic review and meta-analysis. EClinicalMedicine. 2022;48:101458. doi: 10.1016/j.eclinm.2022.101458 35706486 PMC9124702

[37] Bowen AC, Mahé A, Hay RJ, et al. The global epidemiology of impetigo: a systematic review of the population prevalence of impetigo and Pyoderma. PLOS ONE. 2015;10(8):e0136789. doi: 10.1371/journal.pone.0136789 26317533 PMC4552802

[38] Goettsch WG, Bouwes Bavinck JN, Herings RMC. Burden of illness of bacterial cellulitis and erysipelas of the leg in the Netherlands. J Eur Acad Dermatol Venereol. 2006;20(7):834–839. doi: 10.1111/j.1468-3083.2006.01657.x 16898907

[39] Raff AB, Kroshinsky D. Cellulitis: a review. JAMA. 2016;316(3):325–337. doi: 10.1001/jama.2016.8825 27434444

[40] Satoskar AA, Parikh SV, Nadasdy T. Epidemiology, pathogenesis, treatment and outcomes of infection-associated glomerulonephritis. Nat Rev Nephrol. 2020;16(1):32–50. doi: 10.1038/s41581-019-0178-8 31399725

[41] Dunne EM, Hutton S, Peterson E, et al. Increasing incidence of invasive group a streptococcus disease. In: Tony Pearson-Clarke, editor. Emerg. Infect. Dis. Vol. 28. Idaho (USA): Centers for Disease Control and Prevention, *2008-2019*, 2022. p 1785–1795. 35997313 10.3201/eid2809.212129 PMC9423907

[42] Weckel A, Guilbert T, Lambert C, et al. Streptococcus pyogenes infects human endometrium by limiting the innate immune response. J Clin Invest. 2021;131(4). doi: 10.1172/JCI130746 PMC7880408 33320843

[43] Soltani AM, Best MJ, Francis CS, et al. Trends in the incidence and treatment of necrotizing soft tissue infections: an analysis of the national hospital discharge survey. J Burn Care Res. 2014;35:449–454. doi: 10.1097/BCR.0000000000000010 25144805

[44] May AK, Talisa VB, Wilfret DA, et al. Estimating the impact of necrotizing soft tissue infections in the United States: incidence and Re-admissions. Surg Infect. 2021;22(5):509–515. doi: 10.1089/sur.2020.099 32833599

[45] Nanduri SA, Onukwube J, Apostol M, et al. Challenges in surveillance for streptococcal toxic shock syndrome: active bacterial core surveillance, United States, 2014-2017. Public Health Rep. 2022;137(4):687–694. doi: 10.1177/00333549211013460 33960856 PMC9257504

[46] Eriksson BK, Andersson J, Holm SE, et al. Epidemiological and clinical aspects of invasive group a streptococcal infections and the streptococcal toxic shock syndrome. Clin Infect Dis. 1998;27(6):1428–1436. doi: 10.1086/515012 9868656

[47] Vela AI, Villalón P, Sáez-Nieto JA, et al. Characterization of Streptococcus pyogenes from animal clinical specimens, Spain. Emerg Infect Dis. 2017;23(12):2013–2016. doi: 10.3201/eid2312.151146 29148379 PMC5708255

[48] Oliver J, Malliya Wadu E, Pierse N, et al. Group a Streptococcus pharyngitis and pharyngeal carriage: a meta-analysis. PloS Negl Trop Dis. 2018;12(3):e0006335. doi: 10.1371/journal.pntd.0006335 29554121 PMC5875889

[49] Brown WA, Allison VD. Infection of the air of scarlet-fever wards with Streptococcus pyogenes. J Hyg. 1937;37(1):1–13. doi: 10.1017/S0022172400034768 20475359 PMC2199405

[50] Loosli CG, Lemon HM, Wise H, et al. Studies on the transmission and control of respiratory disease within army barracks: I. Hemolytic streptococcal contamination of the environment. J Infect Dis. 1948;82(1):59–71. doi: 10.1093/infdis/82.1.59 18898005

[51] Cordery R, Purba AK, Begum L, et al. Frequency of transmission, asymptomatic shedding, and airborne spread of Streptococcus pyogenes in schoolchildren exposed to scarlet fever: a prospective, longitudinal, multicohort, molecular epidemiological, contact-tracing study in England, UK. The Lancet Microbe. 2022;3(5):e366–e375. doi: 10.1016/S2666-5247(21)00332-3 35544097 PMC9042792

[52] Watts V, Balasegaram S, Brown CS, et al. Increased risk for invasive group a streptococcus disease for household contacts of scarlet fever cases, England, 2011-2016. Emerg Infect Dis. 2019;25:529–537. doi: 10.3201/eid2503.181518 30602121 PMC6390732

[53] Nelson GE, Pondo T, Toews K-A, et al. Epidemiology of invasive group a streptococcal infections in the United States, 2005–2012. Clin Infect Dis. 2016;63(4):478–486. doi: 10.1093/cid/ciw248 27105747 PMC5776658

[54] Carapetis JR, Jacoby P, Carville K, et al. Effectiveness of clindamycin and intravenous immunoglobulin, and risk of disease in contacts, in invasive group a streptococcal infections. Clin Infect Dis. 2014;59(3):358–365. doi: 10.1093/cid/ciu304 24785239

[55] Mearkle R, Balasegaram S, Sriskandan S, et al. Familial transmission of emm12 group a streptococcus. Emerg Infect Dis. 2018;24(11):2133–2134. doi: 10.3201/eid2411.171743 30334731 PMC6200006

[56] Turner CE, Holden MTG, Blane B, et al. The emergence of successful Streptococcus pyogenes lineages through convergent pathways of capsule loss and recombination directing high toxin expression. MBio. 2019), pp. e 10(6):02521–19. doi: 10.1128/mBio.02521-19 PMC6904876 31822586

[57] Nasser W, Beres SB, Olsen RJ, et al. Evolutionary pathway to increased virulence and epidemic group a Streptococcus disease derived from 3,615 genome sequences. Proc Natl Acad Sci USA. 2014), pp. e. 111(17):1768–1776. doi: 10.1073/pnas.1403138111 PMC4035937 24733896

[58] Osowicki J, Nizet V. Malice in chains. J Infect Dis. 2023;227(10):1117–1118. doi: 10.1093/infdis/jiad035 36748315

[59] Lynskey NN, Jauneikaite E, Li HK, et al. Emergence of dominant toxigenic M1T1 Streptococcus pyogenes clone during increased scarlet fever activity in England: a population-based molecular epidemiological study. Lancet Infect Dis. 2019;19(11):1209–1218. doi: 10.1016/S1473-3099(19)30446-3 31519541 PMC6838661

[60] Demczuk W, Martin I, Domingo FR, et al. Identification of Streptococcus pyogenes M1UK clone in Canada. Lancet Infect Dis. 2019;19(12):1284–1285. doi: 10.1016/S1473-3099(19)30622-X 31782392

[61] Li Y, Nanduri SA, Van Beneden CA, et al. M1UK lineage in invasive group a streptococcus isolates from the USA. Lancet Infect Dis. 2020;20(5):538–539. doi: 10.1016/S1473-3099(20)30279-6 PMC9052193 32359463

[62] Moreland NJ, Webb RH. Against the trend: a decrease in scarlet fever in New Zealand. Lancet Infect Dis. 2019;19(12):1285–1286. doi: 10.1016/S1473-3099(19)30617-6 31782393

[63] You Y, Peng X, Yang P, et al. 8-year M type surveillance of Streptococcus pyogenes in China. Lancet Infect Dis. 2020;20(1):24–25. doi: 10.1016/S1473-3099(19)30694-2 31876494

[64] Kotb M, Norrby-Teglund A, McGeer A, et al. An immunogenetic and molecular basis for differences in outcomes of invasive group a streptococcal infections. Nat Med. 2002;8(12):1398–1404. doi: 10.1038/nm1202-800 12436116

[65] Stanevicha V, Eglite J, Sochnevs A, et al. HLA class II associations with rheumatic heart disease among clinically homogeneous patients in children in Latvia. Arthritis Res Ther. 2003;5(6):R340–6. doi: 10.1186/ar1000 14680508 PMC333411

[66] Stanevicha V, Eglite J, Zavadska D, et al. HLA class II DR and DQ genotypes and haplotypes associated with rheumatic fever among a clinically homogeneous patient population of Latvian children. Arthritis Res Ther. 2007;9(3):R58. doi: 10.1186/ar2216 17559688 PMC2206337

[67] Dan JM, Havenar-Daughton C, Kendric K, et al. Recurrent group a streptococcus tonsillitis is an immunosusceptibility disease involving antibody deficiency and aberrant TFH cells. Sci Transl Med. 2019), pp. eaau3776. 11(478). doi: 10.1126/scitranslmed.aau3776 PMC6561727 30728285

[68] Kudat H, Telci G, Sozen AB, et al. The role of HLA molecules in susceptibility to chronic rheumatic heart disease. Int J Immunogenet. 2006;33(1):41–44. doi: 10.1111/j.1744-313X.2006.00562.x 16426242

[69] Parks T, Elliott K, Lamagni T, et al. Elevated risk of invasive group a streptococcal disease and host genetic variation in the human leucocyte antigen locus. Genes Immun. 2020;21(1):63–70. doi: 10.1038/s41435-019-0082-z 31462703 PMC7039814

[70] Imanishi K, Igarashi H, Uchiyama T. Relative abilities of distinct isotypes of human major histocompatibility complex class II molecules to bind streptococcal pyrogenic exotoxin types A and B. Infect Immun. 1992;60(12):5025–5029. doi: 10.1128/iai.60.12.5025-5029.1992 1452333 PMC258272

[71] Llewelyn M, Sriskandan S, Peakman M, et al. HLA class II polymorphisms determine responses to bacterial superantigens. J Immunol. 2004;172(3):1719–1726. doi: 10.4049/jimmunol.172.3.1719 14734754

[72] Kasper KJ, Zeppa JJ, Wakabayashi AT, et al. Bacterial superantigens promote acute nasopharyngeal infection by Streptococcus pyogenes in a human MHC class ii-dependent manner. PloS Pathog. 2014;10(5):e1004155. doi: 10.1371/journal.ppat.1004155 24875883 PMC4038607

[73] Vieira da Silva Pellegrina D, Severino P, Vieira Barbeiro H, et al. Septic shock in advanced age: transcriptome analysis reveals altered molecular signatures in neutrophil granulocytes. PLOS ONE. 2015;10(6):e0128341. doi: 10.1371/journal.pone.0128341 26047321 PMC4457834

[74] Lamb LEM, Sriskandan S, Tan LKK. Bromine, bear-claw scratch fasciotomies, and the Eagle effect: management of group a streptococcal necrotising fasciitis and its association with trauma. Lancet Infect Dis. 2015;15(1):109–121. doi: 10.1016/S1473-3099(14)70922-3 25541175

[75] Langley G, Hao Y, Pondo T, et al. The impact of obesity and diabetes on the risk of disease and death due to invasive group a streptococcus infections in adults. Clin Infect Dis. 2016;62(7):845–852. doi: 10.1093/cid/civ1032 26703865 PMC11331490

[76] Steer AC, Lamagni T, Curtis N, et al. Invasive group a streptococcal disease: epidemiology, pathogenesis and management. Drugs. 2012;72(9):1213–1227. doi: 10.2165/11634180-000000000-00000 22686614 PMC7100837

[77] Laupland KB, Davies HD, Low DE, et al. Invasive group a streptococcal disease in children and association with varicella-zoster virus infection. Ontario group a streptococcal study group. Pediatrics. 2000;105(5):e60–e60. doi: 10.1542/peds.105.5.e60 10799624

[78] Factor SH, Levine OS, Schwartz B, et al. Invasive group a streptococcal disease: risk factors for adults. Emerg Infect Dis. 2003;9(8):970–977. doi: 10.3201/eid0908.020745 12967496 PMC3020599

[79] LaRock CN, Todd J, LaRock DL, et al. IL-1β is an innate immune sensor of microbial proteolysis. Sci Immunol. 2016;1(2):eaah3539. doi: 10.1126/sciimmunol.aah3539 28331908 PMC5358671

[80] Wilde S, Olivares KL, Nizet V, et al. Opportunistic invasive infection by group a streptococcus during anti–interleukin-6 immunotherapy. J Infect Dis. 2021;223(7):1260–1264. doi: 10.1093/infdis/jiaa511 32808035 PMC8030709

[81] Washington A Jr, Varki N, Valderrama JA, et al. Evaluation of IL-17D in host immunity to group a streptococcus infection. J Immunol. 2020;205(11):3122–3129. doi: 10.4049/jimmunol.1901482 33077643 PMC7686091

[82] Bruun T, Rath E, Madsen MB, et al. Risk factors and predictors of mortality in streptococcal necrotizing soft-tissue infections: a multicenter prospective study. Clin Infect Dis. 2021;72(2):293–300. doi: 10.1093/cid/ciaa027 31923305 PMC7840107

[83] Basma H, Norrby-Teglund A, Guedez Y, et al. Risk factors in the pathogenesis of invasive group a streptococcal infections: role of protective humoral immunity. Infect Immun. 1999;67(4):1871–1877. doi: 10.1128/IAI.67.4.1871-1877.1999 10085030 PMC96540

[84] Davies MR, McIntyre L, Mutreja A, et al. Atlas of group a streptococcal vaccine candidates compiled using large-scale comparative genomics. Nat Genet. 2019;51(6):1035–1043. doi: 10.1038/s41588-019-0417-8 31133745 PMC6650292

[85] Steer AC, Law I, Matatolu L, et al. Global emm type distribution of group a streptococci: systematic review and implications for vaccine development. Lancet Infect Dis. 2009;9(10):611–616. doi: 10.1016/S1473-3099(09)70178-1 19778763

[86] Luca-Harari B, Darenberg J, Neal S, et al. Clinical and microbiological characteristics of severe Streptococcus pyogenes disease in Europe. J Clin Microbiol. 2009;47(4):1155–1165. doi:10.1128/JCM.02155-08 19158266 PMC2668334

[87] González-Abad MJ, Alonso Sanz M. Infecciones invasoras por Streptococcus pyogenes (2011-2018): serotipos y presentación clínica. An de Pediatría (English Edition) 2020;92(6):351–358. doi: 10.1016/j.anpedi.2019.10.014 31879253

[88] Yu D, Liang Y, Ma Y, et al. Changes in M types of Streptococcus pyogenes in Chinese children with scarlet fever. Lancet Infect Dis. 2020;20(7):780. doi: 10.1016/S1473-3099(20)30441-2 32592668

[89] Lancefield RC. The antigenic complex of streptococcus haemolyticus. J Exp Med. 1928;47(1):91–103. doi: 10.1084/jem.47.1.91 19869404 PMC2131344

[90] Giannakis E, Sakari Jokiranta T, Ormsby RJ, et al. Identification of the streptococcal M protein binding site on membrane cofactor protein (CD46). The J Immunol. 2002;168(9):4585–4592. doi: 10.4049/jimmunol.168.9.4585 11971006

[91] Cywes C, Wessels MR. Group a streptococcus tissue invasion by CD44-mediated cell signalling. Nature. 2001;414(6864):648–652. doi: 10.1038/414648a 11740562

[92] Cywes C, Stamenkovic I, Wessels MR. CD44 as a receptor for colonization of the pharynx by group a streptococcus. J Clin Invest. 2000;106(8):995–1002. doi: 10.1172/JCI10195 11032859 PMC314343

[93] Schrager HM, Albertí S, Cywes C, et al. Hyaluronic acid capsule modulates M protein-mediated adherence and acts as a ligand for attachment of group a streptococcus to CD44 on human keratinocytes. J Clin Invest. 1998;101(8):1708–1716. doi: 10.1172/JCI2121 9541502 PMC508753

[94] Henningham A, Yamaguchi M, Aziz RK, et al. Mutual exclusivity of hyaluronan and hyaluronidase in invasive group a streptococcus. J Biol Chem. 2014;289(46):32303–32315. doi: 10.1074/jbc.M114.602847 25266727 PMC4231703

[95] Chen Y-H, Li S-H, Yang Y-C, et al. T4 pili promote colonization and immune evasion phenotypes of nonencapsulated M4 Streptococcus pyogenes. MBio. 2020;11(4):01580–01520. doi: 10.1128/mBio.01580-20 PMC7374061 32694142

[96] LaRock DL, Russell R, Johnson AF, et al. Group a streptococcus infection of the nasopharynx requires proinflammatory signaling through the interleukin-1 receptor. Infect Immun. 2020), pp. e. 88(10):00356–20. doi: 10.1128/IAI.00356-20 PMC7504964 32719155

[97] Loh JMS, Rivera-Hernandez T, McGregor R, et al. A multivalent T-antigen-based vaccine for group a streptococcus. Sci Rep. 2021;11(1):4353. doi: 10.1038/s41598-021-83673-4 33623073 PMC7902606

[98] Jespersen MG, Hayes AJ, Tong SYC, et al. Pangenome evaluation of gene essentiality in Streptococcus pyogenes. Microbiol Spectr. 2024;12(8):e0324023. doi: 10.1128/spectrum.03240-23 39012116 PMC11323703

[99] Zhu L, Olsen RJ, Beres SB, et al. Streptococcus pyogenes genes that promote pharyngitis in primates. JCI Insight. 2020;5(11):e137686. doi: 10.1172/jci.insight.137686 32493846 PMC7308061

[100] Zhu L, Olsen RJ, Beres SB, et al. Genome-wide screens identify group a streptococcus surface proteins promoting female genital tract colonization and virulence. Am J Pathol. 2020;190(4):862–873. doi: 10.1016/j.ajpath.2019.12.003 32200972 PMC7184637

[101] Reglinski M. Lancefield whole blood killing assay to evaluate vaccine efficacy. Methods Mol Biol. 2020;2136:317–322. 32430833 10.1007/978-1-0716-0467-0_25

[102] Dale JB, Washburn RG, Marques MB, et al. Hyaluronate capsule and surface M protein in resistance to opsonization of group a streptococci. Infect Immun. 1996;64(5):1495–1501. doi: 10.1128/iai.64.5.1495-1501.1996 8613352 PMC173953

[103] Foley MJ, Wood WB Jr. Studies on the pathogenicity of group a streptococci: II. The antiphagocytic effects of the M protein and the capsular gel. J Exp Med. 1959;110(4):617–628. doi: 10.1084/jem.110.4.617 13823728 PMC2137000

[104] Holm SE, Norrby A, Bergholm A-M, et al. Aspects of pathogenesis of serious group a streptococcal infections in Sweden, 1988–1989. J Infect Dis. 1992;166(1):31–37. doi: 10.1093/infdis/166.1.31 1607705

[105] Whitnack E, Beachey EH. Antiopsonic activity of fibrinogen bound to M protein on the surface of group a streptococci. J Clin Invest. 1982;69(4):1042–1045. doi: 10.1172/JCI110508 7042754 PMC370160

[106] Thern A, Stenberg L, Dahlbäck B, et al. Ig-binding surface proteins of Streptococcus pyogenes also bind human C4b-binding protein (C4BP), a regulatory component of the complement system. The J Immunol. 1995;154(1):375–386. doi: 10.4049/jimmunol.154.1.375 7995956

[107] Pérez-Caballero D, García-Laorden I, Cortés G, et al. Interaction between complement regulators and Streptococcus pyogenes: binding of C4b-binding protein and factor H/factor H-like protein 1 to M18 strains involves two different cell surface molecules. J Immunol. 2004;173(11):6899–6904. doi: 10.4049/jimmunol.173.11.6899 15557185

[108] Carlsson F, Sandin C, Lindahl G. Human fibrinogen bound to Streptococcus pyogenes M protein inhibits complement deposition via the classical pathway. Mol Microbiol. 2005;56(1):28–39. doi: 10.1111/j.1365-2958.2005.04527.x 15773976

[109] Scott JR, Pulliam WM, Hollingshead SK, et al. Relationship of M protein genes in group a streptococci. Proc Natl Acad Sci U S A. 1985;82(6):1822–1826. doi: 10.1073/pnas.82.6.1822 3885219 PMC397365

[110] Bessen D, Jones KF, Fischetti VA. Evidence for two distinct classes of streptococcal M protein and their relationship to rheumatic fever. J Exp Med. 1989;169(1):269–283. doi: 10.1084/jem.169.1.269 2642529 PMC2189173

[111] Cunningham MW, Swerlick RA. Polyspecificity of antistreptococcal murine monoclonal antibodies and their implications in autoimmunity. J Exp Med. 1986;164(4):998–1012. doi: 10.1084/jem.164.4.998 3531385 PMC2188424

[112] Cunningham MW, Antone SM, Gulizia JM, et al. Cytotoxic and viral neutralizing antibodies crossreact with streptococcal M protein, enteroviruses, and human cardiac myosin. Proc Natl Acad Sci U S A. 1992;89(4):1320–1324. doi: 10.1073/pnas.89.4.1320 1311095 PMC48441

[113] Widdowson JP. The M-associated protein antigens of group A streptococci. In: Streptococcal diseases and the immune response. New York, N.Y: Academic Press; 1980. p. 125–147.

[114] Widdowson JP, Maxted WR, Pinney AM. An M-associated protein antigen (MAP) of group a streptococci. Epidemiol Infect. 1971;69(4):553–564. doi: 10.1017/S0022172400021823 PMC2131032 5289717

[115] Pack TD, Boyle MD. Characterization of a type II’o group a streptococcal immunoglobulin-binding protein. Mol Immunol. 1995;32:1235–1243. doi: 10.1016/0161-5890(95)00074-7 8559148

[116] LaRock CN, Döhrmann S, Todd J, et al. Group a streptococcal M1 protein sequesters cathelicidin to evade innate immune killing. Cell Host & Microbe. 2015;18(4):471–477. doi: 10.1016/j.chom.2015.09.004 26468750 PMC4636435

[117] Döhrmann S, LaRock CN, Anderson EL, et al. Group a streptococcal M1 protein provides resistance against the antimicrobial activity of histones. Sci Rep. 2017;7(1):43039. doi: 10.1038/srep43039 28220899 PMC5318940

[118] Buchanan JT, Simpson AJ, Aziz RK, et al. Dnase expression allows the pathogen group a streptococcus to escape killing in neutrophil extracellular traps. Curr Biol. 2006;16(4):396–400. doi: 10.1016/j.cub.2005.12.039 16488874

[119] Uchiyama S, Andreoni F, Schuepbach RA, et al. Dnase Sda1 allows invasive M1T1 group a streptococcus to prevent TLR9-dependent recognition. PloS Pathog. 2012;8(6):e1002736. doi: 10.1371/journal.ppat.1002736 22719247 PMC3375267

[120] Döhrmann S, Anik S, Olson J, et al. Role for streptococcal collagen-like protein 1 in M1T1 group a streptococcus resistance to neutrophil extracellular traps. Infect Immun. 2014;82(10):4011–4020. doi: 10.1128/IAI.01921-14 25024366 PMC4187857

[121] Secundino I, Lizcano A, Roupé KM, et al. Host and pathogen hyaluronan signal through human siglec-9 to suppress neutrophil activation. J Mol Med. 2016;94(2):219–233. doi: 10.1007/s00109-015-1341-8 26411873 PMC4766071

[122] Uchiyama S, Döhrmann S, Timmer AM, et al. Streptolysin O rapidly impairs neutrophil oxidative burst and antibacterial responses to group a streptococcus. Front Immunol. 2015;6:581. doi: 10.3389/fimmu.2015.00581 26635795 PMC4644796

[123] Zimmerman RA, Auernheimer AH, Taranta A. Precipitating antibody to group a streptococcal polysaccharide in humans. J Immunol. 1971;107(3):832–841. doi: 10.4049/jimmunol.107.3.832 4999097

[124] van Sorge NM, Cole JN, Kuipers K, et al. The classical lancefield antigen of group a streptococcus is a virulence determinant with implications for vaccine design. Cell Host & Microbe. 2014;15(6):729–740. doi: 10.1016/j.chom.2014.05.009 24922575 PMC4078075

[125] Henningham A, Davies MR, Uchiyama S, et al. Virulence role of the GlcNAc side chain of the lancefield cell wall carbohydrate antigen in non-M1-serotype group a streptococcus. MBio. 2018), pp. e 9(1):02294–17. doi: 10.1128/mBio.02294-17 PMC5790915 29382733

[126] Chochua S, Rivers J, Mathis S, et al. Emergent invasive group a streptococcus dysgalactiae subsp. equisimilis , United States, 2015–2018. Emerg Infect Dis. 2019;25(8):1543–1547. doi: 10.3201/eid2508.181758 31158071 PMC6649341

[127] Xie O, Zachreson C, Tonkin-Hill G, et al. Overlapping Streptococcus pyogenes and streptococcus dysgalactiae subspecies equisimilis household transmission and mobile genetic element exchange. Nat Commun. 2024;15(1):3477. doi: 10.1038/s41467-024-47816-1 38658529 PMC11043366

[128] Zinkernagel AS, Timmer AM, Pence MA, et al. The IL-8 protease SpyCEP/ScpC of group a streptococcus promotes resistance to neutrophil killing. Cell Host & Microbe. 2008;4(2):170–178. doi: 10.1016/j.chom.2008.07.002 18692776 PMC2631432

[129] Goldblatt J, Lawrenson RA, Muir L, et al. A requirement for neutrophil glycosaminoglycans in Chemokine: Receptor interactions is revealed by the streptococcal protease SpyCEP. J Immunol. 2019;202(11):3246–3255. doi: 10.4049/jimmunol.1801688 31010851 PMC6526389

[130] McKenna S, Malito E, Rouse SL, et al. Structure, dynamics and immunogenicity of a catalytically inactive CXC chemokine-degrading protease SpyCEP from Streptococcus pyogenes. Comput Struct Biotechnol J. 2020;18:650–660. doi: 10.1016/j.csbj.2020.03.004 32257048 PMC7113628

[131] Soderholm AT, Barnett TC, Korn O, et al. Group a streptococcus M1T1 intracellular infection of primary tonsil epithelial cells dampens levels of secreted IL-8 through the action of SpyCEP. Front Cell Infect Microbiol. 2018;8:160. doi: 10.3389/fcimb.2018.00160 29868516 PMC5966554

[132] Lynskey NN, Reglinski M, Calay D, et al. Multi-functional mechanisms of immune evasion by the streptococcal complement inhibitor C5a peptidase. PloS Pathog. 2017;13(8):e1006493. doi: 10.1371/journal.ppat.1006493 28806402 PMC5555575

[133] Park H-S, Cleary PP. Active and passive intranasal immunizations with streptococcal surface protein C5a peptidase prevent infection of murine nasal mucosa-associated lymphoid tissue, a functional homologue of human tonsils. Infect Immun. 2005;73(12):7878–7886. doi: 10.1128/IAI.73.12.7878-7886.2005 16299278 PMC1307028

[134] Wierzbicki IH, Campeau A, Dehaini D, et al. Group a streptococcal S protein utilizes red blood cells as immune camouflage and is a critical determinant for immune evasion. Cell Rep. 2019;29(10):2979–2989.e15. doi: 10.1016/j.celrep.2019.11.001 31801066 PMC6951797

[135] Hertzén E, Johansson L, Wallin R, et al. M1 protein-dependent intracellular trafficking promotes persistence and replication of Streptococcus pyogenes in macrophages. J Innate Immun. 2010;2:534–545. doi: 10.1159/000317635 20798480

[136] Thulin P, Johansson L, Low DE, et al. Viable group a streptococci in macrophages during acute soft tissue infection. PLoS Med. 2006;3(3):e53. doi: 10.1371/journal.pmed.0030053 16401174 PMC1326258

[137] O’Neill AM, Thurston TLM, Holden DW, et al. Cytosolic replication of group a streptococcus in human macrophages. MBio. 2016), pp. e 7(2):00020–16. doi: 10.1128/mBio.00020-16 PMC4959517 27073088

[138] O’Seaghdha M, Wessels MR, Bessen DE. Streptolysin O and its co-toxin nad-glycohydrolase protect group a streptococcus from xenophagic killing. PloS Pathog. 2013;9(6):e1003394. doi: 10.1371/journal.ppat.1003394 23762025 PMC3675196

[139] Lu S-L, Kawabata T, Cheng Y-L, et al. Endothelial cells are intrinsically defective in xenophagy of Streptococcus pyogenes. PloS Pathog. 2017;13(7):e1006444. doi: 10.1371/journal.ppat.1006444 28683091 PMC5500369

[140] Shannon O, Hertzén E, Norrby-Teglund A, et al. Severe streptococcal infection is associated with M protein-induced platelet activation and thrombus formation. Mol Microbiol. 2007;65(5):1147–1157. doi:10.1111/j.1365-2958.2007.05841.x 17662041

[141] Moris V, Guillier D, Zwetyenga N, et al. Streptococcal toxic shock syndrome revealed phlegmasia cerulea dolens of the arm. Lancet Infect Dis. 2020;20(11):1348. doi: 10.1016/S1473-3099(20)30520-X 33098783

[142] Palm F, Sjöholm K, Malmström J, et al. Complement activation occurs at the surface of platelets activated by streptococcal M1 protein and this results in phagocytosis of platelets. J Immunol. 2019;202(2):503–513. doi: 10.4049/jimmunol.1800897 30541884

[143] Palm F, Chowdhury S, Wettemark S, et al. Distinct serotypes of streptococcal M proteins mediate fibrinogen-dependent platelet activation and proinflammatory effects. Infect Immun. 2022;90(2):e0046221. doi: 10.1128/iai.00462-21 34898252 PMC8852700

[144] O’Connor SP, Darip D, Fraley K, et al. The human antibody response to streptococcal C5a peptidase. J Infect Dis. 1991;163(1):109–116. doi: 10.1093/infdis/163.1.109 1984457

[145] Lancefield RC. Persistence of type-specific antibodies in man following infection with group a streptococci. J Exp Med. 1959;110(2):271–292. doi: 10.1084/jem.110.2.271 13673139 PMC2136986

[146] Bencivenga JF, Johnson DR, Kaplan EL. Determination of group a streptococcal anti–M type–specific antibody in sera of rheumatic fever patients after 45 years. Clin Infect Dis. 2009;49(8):1237–1239. doi: 10.1086/605673 19761409

[147] de Neergaard T, Bläckberg A, Ivarsson H, et al. Invasive streptococcal infection can lead to the generation of cross-strain opsonic antibodies. Microbiol Spectr. 2022;10(6):e0248622. doi: 10.1128/spectrum.02486-22 36314947 PMC9769875

[148] Akesson P, Moritz L, Truedsson M, et al. IdeS, a highly specific immunoglobulin G (IgG)-cleaving enzyme from Streptococcus pyogenes, is inhibited by specific IgG antibodies generated during infection. Infect Immun. 2006;74(1):497–503. doi: 10.1128/IAI.74.1.497-503.2006 16369006 PMC1346671

[149] Karlsson CAQ, Järnum S, Winstedt L, et al. Infection and the human proteome with a special focus on the immunoglobulin G-cleaving enzyme IdeS. Mol Cell Proteomics. 2018;17(6):1097–1111. doi: 10.1074/mcp.RA117.000525 29511047 PMC5986240

[150] Collin M, Olsén A. EndoS, a novel secreted protein from Streptococcus pyogenes with endoglycosidase activity on human IgG. Embo J. 2001;20(12):3046–3055. doi: 10.1093/emboj/20.12.3046 11406581 PMC150189

[151] Sudol ASL, Butler J, Ivory DP, et al. Extensive substrate recognition by the streptococcal antibody-degrading enzymes IdeS and EndoS. Nat Commun. 2022;13(1):7801. doi: 10.1038/s41467-022-35340-z 36528711 PMC9759587

[152] Trastoy B, Du JJ, Cifuente JO, et al. Mechanism of antibody-specific deglycosylation and immune evasion by streptococcal IgG-specific endoglycosidases. Nat Commun. 2023;14(1):1705. doi: 10.1038/s41467-023-37215-3 36973249 PMC10042849

[153] Fluckiger U, Jones KF, Fischetti VA. Immunoglobulins to group a streptococcal surface molecules decrease adherence to and invasion of human pharyngeal cells. Infect Immun. 1998;66(3):974–979. doi: 10.1128/IAI.66.3.974-979.1998 9488384 PMC108004

[154] Nordenfelt P, Waldemarson S, Linder A, et al. Antibody orientation at bacterial surfaces is related to invasive infection. J Exp Med. 2012;209(13):2367–2381. doi: 10.1084/jem.20120325 23230002 PMC3526361

[155] Pleass RJ, Areschoug T, Lindahl G, et al. Streptococcal IgA-binding proteins bind in the Cα2-Cα3 interdomain region and inhibit binding of IgA to human CD89. J Biol Chem. 2001;276(11):8197–8204. doi: 10.1074/jbc.M009396200 11096107

[156] Miettinen M, Matikainen S, Vuopio-Varkila J, et al. Lactobacilli and streptococci induce interleukin-12 (IL-12), IL-18, and gamma interferon production in human peripheral blood mononuclear cells. Infect Immun. 1998;66(12):6058–6062. doi: 10.1128/IAI.66.12.6058-6062.1998 9826398 PMC108774

[157] Keller N, Andreoni F, Reiber C, et al. Human streptococcal necrotizing fasciitis histopathology mirrored in a murine Model. Am J Pathol. 2018;188(7):1517–1523. doi: 10.1016/j.ajpath.2018.03.009 29684366

[158] Zhu L, Olsen RJ, Beres SB, et al. Gene fitness landscape of group a streptococcus during necrotizing myositis. J Clin Invest. 2019;129(2):887–901. doi: 10.1172/JCI124994 30667377 PMC6355216

[159] Ayinuola YA, Brito-Robinson T, Ayinuola O, et al. Streptococcus co-opts a conformational lock in human plasminogen to facilitate streptokinase cleavage and bacterial virulence. J Biol Chem. 2021;296:100099. doi: 10.1074/jbc.RA120.016262 33208461 PMC7948469

[160] Ayinuola YA, Tjia-Fleck S, Readnour BM, et al. Relationships between plasminogen-binding M-Protein and surface enolase for human plasminogen acquisition and activation in Streptococcus pyogenes. Front Microbiol. 2022;13:905670. doi: 10.3389/fmicb.2022.905670 35685926 PMC9173704

[161] Sun H, Ringdahl U, Homeister JW, et al. Plasminogen is a critical host pathogenicity factor for group a streptococcal infection. Science. 2004;305(5688):1283–1286. doi:10.1126/science.1101245 15333838

[162] Pancholi V, Fontan P, Jin H. Plasminogen-mediated group a streptococcal adherence to and pericellular invasion of human pharyngeal cells. Microb Pathog. 2003;35(6):293–303. doi: 10.1016/j.micpath.2003.08.004 14580393

[163] Rox K, Jansen R, Loof TG, et al. Linoleic and palmitoleic acid block streptokinase-mediated plasminogen activation and reduce severity of invasive group a streptococcal infection. Sci Rep. 2017;7(1):11798. doi: 10.1038/s41598-017-11276-z 28924140 PMC5603603

[164] Vu HM, Hammers DE, Liang Z, et al. Group a streptococcus-induced activation of human plasminogen is required for keratinocyte wound retraction and rapid clot dissolution. Front Cardiovasc Med. 2021;8:667554. doi: 10.3389/fcvm.2021.667554 34179133 PMC8230121

[165] Chaudhari AM, Vyas S, Singh V, et al. CRISPR-Cas9 mediated knockout of SagD gene for overexpression of streptokinase in streptococcus equisimilis. Microorganisms. 2022;10(3):635. doi: 10.3390/microorganisms10030635 35336210 PMC8953821

[166] Sanderson-Smith ML, Zhang Y, Ly D, et al. A key role for the urokinase plasminogen activator (uPA) in invasive group a streptococcal infection. PloS Pathog. 2013;9(7):e1003469. doi: 10.1371/journal.ppat.1003469 23853591 PMC3701706

[167] Ly D, Donahue D, Walker MJ, et al. Characterizing the role of tissue-type plasminogen activator in a mouse model of group a streptococcal infection. Microbes Infect. 2019;21(8–9):412–417. doi: 10.1016/j.micinf.2019.04.004 31009808 PMC7707001

[168] Olsen RJ, Raghuram A, Cantu C, et al. The majority of 9,729 group a streptococcus strains causing disease secrete SpeB cysteine protease: pathogenesis implications. Infect Immun. 2015;83(12):4750–4758. doi: 10.1128/IAI.00989-15 26416912 PMC4645388

[169] Carothers KE, Liang Z, Mayfield J, et al. The streptococcal protease SpeB antagonizes the biofilms of the human pathogen staphylococcus aureus USA300 through cleavage of the staphylococcal SdrC protein. J Bacteriol. 2020), pp. e. 202(11):00008–20. doi: 10.1128/JB.00008-20 PMC7221255 32205460

[170] Sumitomo T, Mori Y, Nakamura Y, et al. Streptococcal cysteine protease-mediated cleavage of Desmogleins is involved in the pathogenesis of cutaneous infection. Front Cell Infect Microbiol. 2018;8:10. 29416987 10.3389/fcimb.2018.00010 PMC5787553

[171] Egesten A, Olin AI, Linge HM, et al. SpeB of Streptococcus pyogenes differentially modulates antibacterial and receptor activating properties of human chemokines. PLoS One. 2009;4(3):e4769. doi: 10.1371/journal.pone.0004769 19274094 PMC2652026

[172] Edwards RJ, Pyzio M, Gierula M, et al. Proteomic analysis at the sites of clinical infection with invasive Streptococcus pyogenes. Sci Rep. 2018;8(1):5950. doi: 10.1038/s41598-018-24216-2 29654237 PMC5899161

[173] Tsai PJ, Lin YS, Kuo CF, et al. Group a Streptococcus induces apoptosis in human epithelial cells. Infect Immun. 1999;67(9):4334–4339. doi: 10.1128/IAI.67.9.4334-4339.1999 10456871 PMC96749

[174] Deng W, Bai Y, Deng F, et al. Streptococcal pyrogenic exotoxin B cleaves GSDMA and triggers pyroptosis. Nature. 2022;602(7897):496–502. doi: 10.1038/s41586-021-04384-4 35110732 PMC9703647

[175] LaRock DL, Johnson AF, Wilde S, et al. Group a streptococcus induces gsdma-dependent pyroptosis in keratinocytes. Nature. 2022;605(7910):527–531. doi: 10.1038/s41586-022-04717-x 35545676 PMC9186297

[176] Woehl JL, Kitamura S, Dillon N, et al. An irreversible inhibitor to probe the role of Streptococcus pyogenes cysteine protease SpeB in evasion of Host complement defenses. ACS Chem Biol. 2020;15(8):2060–2069. doi: 10.1021/acschembio.0c00191 32662975 PMC7755099

[177] Walker MJ, Hollands A, Sanderson-Smith ML, et al. Dnase Sda1 provides selection pressure for a switch to invasive group a streptococcal infection. Nat Med. 2007;13(8):981–985. doi: 10.1038/nm1612 17632528

[178] Afshar B, Turner CE, Lamagni TL, et al. Enhanced nasopharyngeal infection and shedding associated with an epidemic lineage of emm3 group a streptococcus. Virulence. 2017;8(7):1390–1400. doi: 10.1080/21505594.2017.1325070 28459299 PMC5711448

[179] Lynskey NN, Banerji S, Johnson LA, et al. Rapid lymphatic dissemination of encapsulated group a streptococci via lymphatic vessel endothelial receptor-1 interaction. PloS Pathog. 2015;11(9):e1005137. doi: 10.1371/journal.ppat.1005137 26352587 PMC4564194

[180] Siggins MK, Lynskey NN, Lamb LE, et al. Extracellular bacterial lymphatic metastasis drives Streptococcus pyogenes systemic infection. Nat Commun. 2020;11(1):4697. doi: 10.1038/s41467-020-18454-0 32943639 PMC7498588

[181] Lamb LE, Siggins MK, Scudamore C, et al. Impact of contusion injury on intramuscular emm1 group a streptococcus infection and lymphatic spread. Virulence. 2018;9(1):1074–1084. doi: 10.1080/21505594.2018.1482180 30052105 PMC6068544

[182] Hoe NP, Kordari P, Cole R, et al. Human immune response to streptococcal inhibitor of complement, a serotype M1 group a Streptococcus extracellular protein involved in epidemics. J Infect Dis. 2000;182(5):1425–1436. doi: 10.1086/315882 11015234

[183] Tan LKK, Reglinski M, Teo D, et al. Vaccine-induced, but not natural immunity, against the streptococcal inhibitor of complement protects against invasive disease. NPJ Vaccines. 2021;6(1):62. doi: 10.1038/s41541-021-00326-3 33888727 PMC8062509

[184] Breiman RF, Davis JP, Facklam RR, et al. Defining the group a streptococcal toxic shock syndrome: rationale and consensus definition. JAMA. 1993;269(3):390–391. doi:10.1001/jama.1993.03500030088038 8418347

[185] Tuffs SW, Dufresne K, Rishi A, et al. Novel insights into the immune response to bacterial T cell superantigens. Nat Rev Immunol. 2024;24(6):417–434. doi: 10.1038/s41577-023-00979-2 38225276

[186] Timmer AM, Timmer JC, Pence MA, et al. Streptolysin O promotes group a streptococcus immune evasion by accelerated macrophage apoptosis. J Biol Chem. 2009;284(2):862–871. doi: 10.1074/jbc.M804632200 19001420 PMC2613605

[187] Shewell LK, Day CJ, Jen FE-C, et al. All major cholesterol-dependent cytolysins use glycans as cellular receptors. Sci Adv. 2020;6(21):eaaz4926. doi:10.1126/sciadv.aaz4926 32494740 PMC7244308

[188] Bryant AE, Bayer CR, Chen RYZ, et al. Vascular dysfunction and ischemic destruction of tissue in Streptococcus pyogenes infection: the role of streptolysin O–induced Platelet/Neutrophil complexes. J Infect Dis. 2005;192(6):1014–1022. doi: 10.1086/432729 16107954

[189] Higashi DL, Biais N, Donahue DL, et al. Activation of band 3 mediates group a streptococcus streptolysin S-based beta-haemolysis. Nat Microbiol. 2016;1(2):15004. doi: 10.1038/nmicrobiol.2015.4 27571972

[190] Hammers DE, Donahue DL, Tucker ZD, et al. Streptolysin S targets the sodium-bicarbonate cotransporter NBCn1 to induce inflammation and cytotoxicity in human keratinocytes during group a streptococcal infection. Front Cell Infect Microbiol. 2022;12:1002230. doi: 10.3389/fcimb.2022.1002230 36389147 PMC9663810

[191] Shannon BA, Hurst JR, Flannagan RS, et al. Streptolysin S is required for Streptococcus pyogenes nasopharyngeal and skin infection in hla-transgenic mice. PloS Pathog. 2024;20(3):e1012072. doi: 10.1371/journal.ppat.1012072 38452154 PMC10950238

[192] Pinho-Ribeiro FA, Baddal B, Haarsma R, et al. Blocking neuronal signaling to immune cells treats streptococcal invasive infection. Cell. 2018;173(5):1083–1097.e22. doi: 10.1016/j.cell.2018.04.006 29754819 PMC5959783

[193] Reglinski M, Sriskandan S, Turner CE. Identification of two new core chromosome-encoded superantigens in Streptococcus pyogenes; speQ and speR. J Infect. 2019;78(5):358–363. doi: 10.1016/j.jinf.2019.02.005 30796950

[194] Bergsten H, Madsen MB, Bergey F, et al. Correlation between immunoglobulin dose administered and plasma neutralization of streptococcal superantigens in patients with necrotizing soft tissue infections. Clin Infect Dis. 2020;71(7):1772–1775. doi: 10.1093/cid/ciaa022 31916575

[195] Chatellier S, Ihendyane N, Kansal RG, et al. Genetic relatedness and superantigen expression in group a streptococcus serotype M1 isolates from patients with severe and nonsevere invasive diseases. Infect Immun. 2000;68(6):3523–3534. doi: 10.1128/IAI.68.6.3523-3534.2000 10816507 PMC97638

[196] Norrby-Teglund A, Chatellier S, Low DE, et al. Host variation in cytokine responses to superantigens determine the severity of invasive group a streptococcal infection. Eur J Immunol. 2000;30(11):3247–3255. doi: 10.1002/1521-4141(200011)30:11<3247::AID-IMMU3247>3.0.CO;2-D 11093140

[197] Zeppa JJ, Kasper KJ, Mohorovic I, et al. Nasopharyngeal infection by Streptococcus pyogenes requires superantigen-responsive Vβ-specific T cells. Proc Natl Acad Sci U S A. 2017;114(38):10226–10231. doi: 10.1073/pnas.1700858114 28794279 PMC5617250

[198] Thomas D, Dauwalder O, Brun V, et al. Staphylococcus aureus superantigens elicit redundant and extensive human Vβ patterns. Infection. 2009;77(5):2043–2050. doi: 10.1128/IAI.01388-08 PMC2681734 19255190

[199] Emgård J, Bergsten H, McCormick JK, et al. MAIT cells are major contributors to the cytokine response in group a streptococcal toxic shock syndrome. Proc Natl Acad Sci U S A. 2019;116(51):25923–25931. doi: 10.1073/pnas.1910883116 31772015 PMC6926028

[200] Davies FJ, Olme C, Lynskey NN, et al. Streptococcal superantigen-induced expansion of human tonsil T cells leads to altered T follicular helper cell phenotype, B cell death and reduced immunoglobulin release. Clin & Exp Immunol. 2019;197(1):83–94. doi: 10.1111/cei.13282 30815853 PMC6591145

[201] Johnson DA, Piper IM, Vogel BA, et al. Structures of Streptococcus pyogenes class a sortase in complex with substrate and product mimics provide key details of target recognition. J Biol Chem. 2022;298(10):102446. doi: 10.1016/j.jbc.2022.102446 36055407 PMC9520033

[202] Påhlman LI, Olin AI, Darenberg J, et al. Soluble M1 protein of Streptococcus pyogenes triggers potent T cell activation. Cell Microbiol. 2008:404–414. doi:10.1111/j.1462-5822.2007.01053.x 17900297

[203] Valderrama JA, Riestra AM, Gao NJ, et al. Group a streptococcal M protein activates the NLRP3 inflammasome. Nat Microbiol. 2017;2(10):1425–1434. doi: 10.1038/s41564-017-0005-6 28784982 PMC5750061

[204] Gerber MA, Tanz RR, Kabat W, et al. Potential mechanisms for failure to eradicate group a streptococci from the pharynx. Pediatrics. 1999;104(4):911–917. doi: 10.1542/peds.104.4.911 10506234

[205] Li A, Wang N, Ge L, et al. Risk factors of recurrent erysipelas in adult Chinese patients: a prospective cohort study. BMC Infect Dis. 2021;21(1):26. doi: 10.1186/s12879-020-05710-3 33413190 PMC7792156

[206] LaPenta D, Rubens C, Chi E, et al. Group a streptococci efficiently invade human respiratory epithelial cells. Proc Natl Acad Sci U S A. 1994;91(25):12115–12119. doi: 10.1073/pnas.91.25.12115 7991594 PMC45387

[207] Osterlund A, Popa R, Nikkilä T, et al. Intracellular reservoir of Streptococcus pyogenes in vivo: a possible explanation for recurrent pharyngotonsillitis. Laryngoscope. 1997;107(5):640–647. doi: 10.1097/00005537-199705000-00016 9149167

[208] Baldassarri L, Creti R, Imperi M, et al. Detection of genes encoding internalization-associated proteins in Streptococcus pyogenes isolates from patients with invasive diseases and asymptomatic carriers. J Clin Microbiol. 2007;45(4):1284–1287. doi: 10.1128/JCM.02119-06 17287324 PMC1865825

[209] Siemens N, Chakrakodi B, Shambat SM, et al. Biofilm in group a streptococcal necrotizing soft tissue infections. JCI Insight. 2016;1(10):e87882. doi: 10.1172/jci.insight.87882 27699220 PMC5033946

[210] Matysik A, Ho FK, Ler Tan AQ, et al. Cellular chaining influences biofilm formation and structure in group a streptococcus. Biofilms. 2020;2:100013. doi: 10.1016/j.bioflm.2019.100013 PMC7798446 33447800

[211] Wang B, Dileepan T, Briscoe S, et al. Induction of tgf-β1 and tgf-β1–dependent predominant Th17 differentiation by group A streptococcal infection. 2010;107:5937–5942. 10.1073/pnas.0904831107 PMC2851870 20231435

[212] Courtney HS, Hasty DL. Aggregation of group a streptococci by human saliva and effect of saliva on streptococcal adherence to host cells. Infect Immun. 1991;59(5):1661–1666. doi:10.1128/iai.59.5.1661-1666.1991 2019436 PMC257899

[213] Cho KH, Port GC, Caparon M, et al. Genetics of group a streptococci. Microbiol Spectr. 2019;7(2):10.1128. doi:10.1128/microbiolspec.GPP3-0056-2018 PMC11590684 30825299

[214] Kreikemeyer B, McIver KS, Podbielski A. Virulence factor regulation and regulatory networks in Streptococcus pyogenes and their impact on pathogen–host interactions. Trends Microbiol. 2003;11(5):224–232. doi: 10.1016/S0966-842X(03)00098-2 12781526

[215] Luo F, Lizano S, Bessen DE. Heterogeneity in the polarity of nra regulatory effects on streptococcal pilus gene transcription and virulence. Infect Immun. 2008;76(6):2490–2497. doi: 10.1128/IAI.01567-07 18347035 PMC2423052

[216] Finn MB, Ramsey KM, Dove SL, et al. Identification of group a streptococcus genes directly regulated by CsrRS and novel intermediate regulators. MBio. 2021;12(4):e0164221. doi: 10.1128/mBio.01642-21 34253064 PMC8406183

[217] Voyich JM, Sturdevant DE, Braughton KR, et al. Genome-wide protective response used by group a streptococcus to evade destruction by human polymorphonuclear leukocytes. Proc Natl Acad Sci U S A. 2003;100(4):1996–2001. doi:10.1073/pnas.0337370100 12574517 PMC149947

[218] Voyich JM, Braughton KR, Sturdevant DE, et al. Engagement of the pathogen survival response used by group a streptococcus to avert destruction by innate host defense. J Immunol. 2004;173(2):1194–1201. doi:10.4049/jimmunol.173.2.1194 15240710

[219] Hertzén E, Johansson L, Kansal R, et al. Intracellular Streptococcus pyogenes in human macrophages display an altered gene expression profile. PLOS ONE. 2012;7(4):e35218. doi: 10.1371/journal.pone.0035218 22511985 PMC3325220

[220] Hertzog BB, Kaufman Y, Biswas D, et al. A sub-population of group a streptococcus elicits a population-wide production of bacteriocins to establish dominance in the host. Cell Host & Microbe. 2018;23(3):312–323.e6. doi: 10.1016/j.chom.2018.02.002 29544095

[221] Anand A, Sharma A, Ravins M, et al. Unfolded protein response inhibitors cure group a streptococcal necrotizing fasciitis by modulating host asparagine. Sci Transl Med. 2021;13(605):eabd7465. doi: 10.1126/scitranslmed.abd7465 34349034

[222] Hirose Y, Yamaguchi M, Sumitomo T, et al. Streptococcus pyogenes upregulates arginine catabolism to exert its pathogenesis on the skin surface. Cell Rep. 2021;34(13):108924. doi: 10.1016/j.celrep.2021.108924 33789094 PMC9214650

[223] Banerji R, Saroj SD. Interspecies signaling affects virulence related morphological characteristics of Streptococcus pyogenes M3. FEMS Microbiol Lett. 2021;368(13):fnab079. doi: 10.1093/femsle/fnab079 34156082

[224] Deltcheva E, Chylinski K, Sharma CM, et al. CRISPR RNA maturation by trans-encoded small RNA and host factor RNase III. Nature. 2011;471(7340):602–607. doi: 10.1038/nature09886 21455174 PMC3070239

[225] Chen JS, Dagdas YS, Kleinstiver BP, et al. Enhanced proofreading governs crispr–Cas9 targeting accuracy. Nature. 2017;550(7676):407–410. doi: 10.1038/nature24268 28931002 PMC5918688

[226] Hu JH, Miller SM, Geurts MH, et al. Evolved Cas9 variants with broad PAM compatibility and high DNA specificity. Nature. 2018;556(7699):57–63. doi: 10.1038/nature26155 29512652 PMC5951633

[227] Gao NJ, Al-Bassam MM, Poudel S, et al. Functional and proteomic analysis of Streptococcus pyogenes virulence upon loss of its native Cas9 nuclease. Front Microbiol. 2019;10:1967. doi: 10.3389/fmicb.2019.01967 31507572 PMC6714885

[228] Charlesworth CT, Deshpande PS, Dever DP, et al. Identification of preexisting adaptive immunity to Cas9 proteins in humans. Nat Med. 2019;25(2):249–254. doi: 10.1038/s41591-018-0326-x 30692695 PMC7199589

[229] Wagner DL, Amini L, Wendering DJ, et al. High prevalence of Streptococcus pyogenes Cas9-reactive T cells within the adult human population. Nat Med. 2019;25(2):242–248. doi: 10.1038/s41591-018-0204-6 30374197

[230] Liao C, Sharma S, Svensson SL, et al. Spacer prioritization in crispr–Cas9 immunity is enabled by the leader RNA. Nat Microbiol. 2022;7(4):530–541. doi: 10.1038/s41564-022-01074-3 35314780 PMC7612570

[231] Varble A, Campisi E, Euler CW, et al. Prophage integration into CRISPR loci enables evasion of antiviral immunity in Streptococcus pyogenes. Nat Microbiol. 2021;6(12):1516–1525. doi: 10.1038/s41564-021-00996-8 34819640

[232] Hynes AP, Rousseau GM, Lemay M-L, et al. An anti-crispr from a virulent streptococcal phage inhibits Streptococcus pyogenes Cas9. Nat Microbiol. 2017;2(10):1374–1380. doi: 10.1038/s41564-017-0004-7 28785032

[233] Wong C-H, Khin L-W, Heng K-S, et al. The LRINEC (laboratory risk indicator for necrotizing fasciitis) score: a tool for distinguishing necrotizing fasciitis from other soft tissue infections. Crit Care Med. 2004;32(7):1535–1541. doi: 10.1097/01.CCM.0000129486.35458.7D 15241098

[234] Hansen MB, Rasmussen LS, Svensson M, et al. Association between cytokine response, the LRINEC score and outcome in patients with necrotising soft tissue infection: a multicentre, prospective study. Sci Rep. 2017;7(1):42179. doi: 10.1038/srep42179 28176831 PMC5296880

[235] Borschitz T, Schlicht S, Siegel E, et al. Improvement of a clinical score for necrotizing fasciitis: ‘pain out of proportion’ and high CRP levels aid the diagnosis. PLOS ONE. 2015;10(7):e0132775. doi: 10.1371/journal.pone.0132775 26196941 PMC4511009

[236] Gazzano V, Berger A, Benito Y, et al. Reassessment of the role of rapid antigen detection tests in diagnosis of invasive group a streptococcal infections. J Clin Microbiol. 2016;54(4):994–999. doi: 10.1128/JCM.02516-15 26818671 PMC4809959

[237] Luo R, Sickler J, Vahidnia F, et al. Diagnosis and management of group a streptococcal pharyngitis in the United States, 2011–2015. BMC Infect Dis. 2019;19(1):193. doi: 10.1186/s12879-019-3835-4 30808305 PMC6390592

[238] Hansen MB, Rasmussen LS, Garred P, et al. Associations of plasma nitrite, L-Arginine and asymmetric dimethylarginine with morbidity and mortality in patients with necrotizing soft tissue infections. Shock. 2018;49(6):667–674. doi: 10.1097/SHK.0000000000000975 28863028 PMC5929495

[239] Hansen MB, Rasmussen LS, Garred P, et al. Pentraxin-3 as a marker of disease severity and risk of death in patients with necrotizing soft tissue infections: a nationwide, prospective, observational study. Crit Care. 2016;20(1):40. doi: 10.1186/s13054-016-1210-z 26880104 PMC4754810

[240] Medina LMP, Rath E, Jahagirdar S, et al. Discriminatory plasma biomarkers predict specific clinical phenotypes of necrotizing soft-tissue infections. J Clin Invest. 2021 7;131(14):e149523. doi: 10.1172/JCI149523 34263738 PMC8279592

[241] Thänert R, Itzek A, Hoßmann J, et al. Molecular profiling of tissue biopsies reveals unique signatures associated with streptococcal necrotizing soft tissue infections. Nat Commun. 2019;10(1):3846. doi: 10.1038/s41467-019-11722-8 31451691 PMC6710258

[242] Sjöholm K, Karlsson C, Linder A, et al. A comprehensive analysis of the Streptococcus pyogenes and human plasma protein interaction network. Mol BioSyst. 2014;10(7):1698–1708. doi: 10.1039/C3MB70555B 24525632

[243] Lapek JD Jr, Mills RH, Wozniak JM, et al. Defining host responses during systemic bacterial infection through construction of a murine organ proteome atlas. Cell Syst. 2018;6(5):579–592.e4. doi: 10.1016/j.cels.2018.04.010 29778837 PMC7868092

[244] Andreoni F, Zürcher C, Tarnutzer A, et al. Clindamycin affects group a streptococcus virulence factors and improves clinical outcome. J Infect Dis. 2017;215:269–277. doi: 10.1093/infdis/jiw229 27247345

[245] Babiker A, Li X, Lai YL, et al. Effectiveness of adjunctive clindamycin in β-lactam antibiotic-treated patients with invasive β-haemolytic streptococcal infections in US hospitals: a retrospective multicentre cohort study. Lancet Infect Dis. 2021;21(5):697–710. doi: 10.1016/S1473-3099(20)30523-5 33333013 PMC8084921

[246] Norrby-Teglund A, Kaul R, Low DE, et al. Evidence for the presence of streptococcal-superantigen-neutralizing antibodies in normal polyspecific immunoglobulin G. Infect Immun. 1996;64(12):5395–5398. doi:10.1128/iai.64.12.5395-5398.1996 8945593 PMC174535

[247] Darenberg J, Ihendyane N, Sjölin J, et al. Intravenous immunoglobulin G therapy in streptococcal toxic shock syndrome: a European randomized, double-blind, placebo-controlled trial. Clin Infect Dis. 2003;37(3):333–340. doi: 10.1086/376630 12884156

[248] Linnér A, Darenberg J, Sjölin J, et al. Clinical efficacy of polyspecific intravenous immunoglobulin therapy in patients with streptococcal toxic shock syndrome: a comparative observational study. Clin Infect Dis. 2014;59(6):851–857. doi: 10.1093/cid/ciu449 24928291

[249] Norrby-Teglund A, Muller MP, McGeer A, et al. Successful management of severe group a streptococcal soft tissue infections using an aggressive medical regimen including intravenous polyspecific immunoglobulin together with a conservative surgical approach. Scand J Infect Dis. 2005;37(3):166–172. doi: 10.1080/00365540410020866 15849047

[250] Madsen MB, Hjortrup PB, Hansen MB, et al. Immunoglobulin G for patients with necrotising soft tissue infection (INSTINCT): a randomised, blinded, placebo-controlled trial. Intensive Care Med. 2017;43(11):1585–1593. doi: 10.1007/s00134-017-4786-0 28421246

[251] Parks T, Wilson C, Curtis N, et al. Polyspecific intravenous immunoglobulin in clindamycin-treated patients with streptococcal toxic shock syndrome: a systematic review and meta-analysis. Clin Infect Dis. 2018;67(9):1434–1436. doi: 10.1093/cid/ciy401 29788397 PMC6186853

[252] Reglinski M, Sriskandan S. Treatment potential of pathogen-reactive antibodies sequentially purified from pooled human immunoglobulin. BMC Res Notes. 2019;12(1):228. doi: 10.1186/s13104-019-4262-8 30992057 PMC6466806

[253] Levett D, Bennett MH, Millar I. Adjunctive hyperbaric oxygen for necrotizing fasciitis. Cochrane Database Syst Rev. 2015), pp. CD007937. 1(12). doi: 10.1002/14651858.CD007937.pub2 PMC6516968 25879088

[254] Hedetoft M, Bennett MH, Hyldegaard O. Adjunctive hyperbaric oxygen treatment for necrotising soft-tissue infections: a systematic review and meta-analysis. Diving Hyperb Med. 2021;51(1):34–43. doi: 10.28920/dhm51.1.34-43 33761539 PMC8081587

[255] Mladenov A, Diehl K, Müller O, et al. Outcome of necrotizing fasciitis and Fournier’s gangrene with and without hyperbaric oxygen therapy: a retrospective analysis over 10 years. World J Emerg Surg. 2022;17(1):43. doi: 10.1186/s13017-022-00448-6 35932075 PMC9356491

[256] Toppen W, Cho NY, Sareh S, et al. Contemporary national outcomes of hyperbaric oxygen therapy in necrotizing soft tissue infections. PLOS ONE. 2024;19(3):e0300738. doi: 10.1371/journal.pone.0300738 38512943 PMC10956790

[257] Alphonse MP, Rubens JH, Ortines RV, et al. Pan-caspase inhibition as a potential host-directed immunotherapy against MRSA and other bacterial skin infections. Sci Transl Med. 2021;13(601):eabe9887. doi: 10.1126/scitranslmed.abe9887 34233954 PMC8665304

[258] Chen Y, Chen M, Zhang Y, et al. Broad‐Spectrum neutralization of Pore‐Forming toxins with human erythrocyte Membrane‐Coated nanosponges. Adv Healthc Mater. 2018;7(13):1701366. doi:10.1002/adhm.201701366 PMC6041168 29436150

[259] Herrera AL, Van Hove C, Hanson M, et al. Immunotherapy targeting the Streptococcus pyogenes M protein or streptolysin O to treat or prevent influenza a superinfection. PLOS ONE. 2020;15(6):e0235139. doi: 10.1371/journal.pone.0235139 32574205 PMC7310742

[260] Horn DL, Roberts EA, Shen J, et al. Outcomes of β-hemolytic streptococcal necrotizing skin and soft-tissue infections and the impact of clindamycin resistance. Clin Infect Dis. 2021;73(11):e4592–e4598. doi: 10.1093/cid/ciaa976 33151283 PMC8664434

[261] Cortés-Penfield N, Ryder JH. Should linezolid replace clindamycin as the adjunctive antimicrobial of choice in group a streptococcal necrotizing soft tissue infection and toxic shock syndrome? A focused debate. Clin Infect Dis. 2022;76(2):346–350. doi: 10.1093/cid/ciac720 36056891

[262] Bryant AE, Bayer CR, Aldape MJ, et al. Emerging erythromycin and clindamycin resistance in group a streptococci: efficacy of linezolid and tedizolid in experimental necrotizing infection. J Glob Antimicrob Resist. 2020;22:601–607. doi: 10.1016/j.jgar.2020.04.032 32408046

[263] Bergsten H, Palma Medina LM, Morgan M, et al. Adjunctive rifampicin increases antibiotic efficacy in group a streptococcal tissue infection models. Antimicrob Agents Chemother. 2021;65(11):e0065821. doi:10.1128/AAC.00658-21 34491807 PMC8522778

[264] Vannice KS, Ricaldi J, Nanduri S, et al. Streptococcus pyogenes pbp2x mutation confers reduced susceptibility to β-lactam antibiotics. Clin Infect Dis. 2020;71(1):201–204. doi: 10.1093/cid/ciz1000 31630171 PMC7167332

[265] Musser JM, Beres SB, Zhu L, et al. Reduced in vitro susceptibility of Streptococcus pyogenes to β-lactam antibiotics associated with mutations in the pbp2x gene is geographically widespread. J Clin Microbiol. 2020), pp. e. 58(4):01993–19. doi: 10.1128/JCM.01993-19 PMC7098749 31996443

[266] Yang P, Peng X, Zhang D, et al. Characteristics of group a streptococcus strains circulating during scarlet fever epidemic. In: Emerg. Infect. Dis. Vol. 19. Beijing, China: Centers for Disease Control and Prevention; 2013. p 909–915. 23735582 10.3201/eid1906.121020 PMC4816378

[267] Ayer V, Tewodros W, Manoharan A, et al. Tetracycline resistance in group a streptococci: emergence on a global scale and influence on multiple-drug resistance. Antimicrob Agents Chemother. 2007;51(5):1865–1868. doi: 10.1128/AAC.01341-06 17307980 PMC1855555

[268] Montes M, Tamayo E, Orden B, et al. Prevalence and clonal characterization of Streptococcus pyogenes clinical isolates with reduced fluoroquinolone susceptibility in Spain. Antimicrob Agents Chemother. 2010;54(1):93–97. doi: 10.1128/AAC.00780-09 19805559 PMC2798512

[269] Vekemans J, Gouvea-Reis F, Kim JH, et al. The path to group a streptococcus vaccines: world health organization research and development technology roadmap and preferred product characteristics. Clin Infect Dis. 2019;69(5):877–883. doi: 10.1093/cid/ciy1143 30624673 PMC6695511

[270] Walkinshaw DR, Wright MEE, Mullin AE, et al. The Streptococcus pyogenes vaccine landscape. NPJ Vaccines. 2023;8(1):16. doi: 10.1038/s41541-023-00609-x 36788225 PMC9925938

[271] Kotloff KL, Corretti M, Palmer K, et al. Safety and immunogenicity of a recombinant multivalent group a streptococcal vaccine in healthy adults: phase 1 trial. JAMA. 2004;292(6):709–715. doi: 10.1001/jama.292.6.709 15304468

[272] McNeil SA, Halperin SA, Langley JM, et al. Safety and immunogenicity of 26-valent group a streptococcus vaccine in healthy adult volunteers. Clin Infect Dis. 2005;41(8):1114–1122. doi: 10.1086/444458 16163629

[273] Pastural É, McNeil SA, MacKinnon-Cameron D, et al. Safety and immunogenicity of a 30-valent M protein-based group a streptococcal vaccine in healthy adult volunteers: a randomized, controlled phase I study. Vaccine. 2020;38(6):1384–1392. doi: 10.1016/j.vaccine.2019.12.005 31843270

[274] Sekuloski S, Batzloff MR, Griffin P, et al. Evaluation of safety and immunogenicity of a group a streptococcus vaccine candidate (MJ8VAX) in a randomized clinical trial. PLoS One. 2018;13(7):e0198658. doi: 10.1371/journal.pone.0198658 29965967 PMC6028081

[275] Rivera-Hernandez T, Carnathan DG, Jones S, et al. An experimental group a streptococcus vaccine that reduces pharyngitis and tonsillitis in a nonhuman primate model. MBio. 2019), pp. e 10(2):00693–19. doi: 10.1128/mBio.00693-19 PMC6495378 31040243

[276] Gao NJ, Uchiyama S, Pill L, et al. Site-specific conjugation of cell wall polyrhamnose to protein SpyAD envisioning a safe universal group a streptococcal vaccine. Infect Microbes & Dis. 2021;3(2):87. doi: 10.1097/IM9.0000000000000044 39450141 PMC11501091

[277] Kapoor N, Uchiyama S, Pill L, et al. Non-native amino acid click chemistry-based technology for site-specific polysaccharide conjugation to a bacterial protein serving as both carrier and vaccine antigen. ACS Omega. 2022;7(28):24111–24120. doi:10.1021/acsomega.1c07360 35874267 PMC9301713

[278] Di Benedetto R, Mancini F, Carducci M, et al. Rational design of a glycoconjugate vaccine against group a streptococcus. Int J Mol Sci. 2020;21(22):8558. doi: 10.3390/ijms21228558 33202815 PMC7696035

[279] Pandey M, Calcutt A, Ozberk V, et al. Antibodies to the conserved region of the M protein and a streptococcal superantigen cooperatively resolve toxic shock-like syndrome in hla-humanized mice. Sci Adv. 2019;5(9):eaax3013. doi:10.1126/sciadv.aax3013 31517054 PMC6726444

[280] Jones S, Moreland NJ, Zancolli M, et al. Development of an opsonophagocytic killing assay for group a streptococcus. Vaccine. 2018;36(26):3756–3763. doi: 10.1016/j.vaccine.2018.05.056 29776751

[281] Shaw HA, Ozanne J, Burns K, et al. Multicomponent vaccines against group a streptococcus can effectively target broad disease presentations. Vaccines (Basel). 2021;9(9):1025. doi: 10.3390/vaccines9091025 34579262 PMC8473114

[282] Osowicki J, Azzopardi KI, Fabri L, et al. A controlled human infection model of Streptococcus pyogenes pharyngitis (CHIVAS-M75): an observational, dose-finding study. The Lancet Microbe. 2021;2(7):e291–e299. doi: 10.1016/S2666-5247(20)30240-8 35544165

[283] Bennett J, Moreland NJ, Zhang J, et al. Risk factors for group a streptococcal pharyngitis and skin infections: a case control study. The Lancet Reg Health - West Pac. 2022;26:100507. doi: 10.1016/j.lanwpc.2022.100507 35789826 PMC9250036

[284] Bryant AE, Hayes-Schroer SM, Stevens DL. M type 1 and 3 group a streptococci stimulate tissue factor-mediated procoagulant activity in human monocytes and endothelial cells. Infect Immun. 2003;71(4):1903–1910. doi: 10.1128/IAI.71.4.1903-1910.2003 12654807 PMC152020

[285] Cole JN, Aziz RK, Kuipers K, et al. A conserved udp-glucose dehydrogenase encoded outside the hasABC operon contributes to capsule biogenesis in group a streptococcus. J Bacteriol. 2012;194(22):6154–6161. doi: 10.1128/JB.01317-12 22961854 PMC3486382

[286] Kachroo P, Eraso JM, Beres SB, et al. Integrated analysis of population genomics, transcriptomics and virulence provides novel insights into Streptococcus pyogenes pathogenesis. Nat Genet. 2019;51(3):548–559. doi: 10.1038/s41588-018-0343-1 30778225 PMC8547240

